# Spatio-temporal characterization of the antiviral activity of the XRN1-DCP1/2 aggregation against cytoplasmic RNA viruses to prevent cell death

**DOI:** 10.1038/s41418-020-0509-0

**Published:** 2020-02-07

**Authors:** Chen Seng Ng, Dacquin M. Kasumba, Takashi Fujita, Honglin Luo

**Affiliations:** 1 Centre for Heart Lung Innovation, St. Paul’s Hospital, University of British Columbia, Vancouver, BC Canada; 2 Department of Pathology and Laboratory Medicine, University of British Columbia, Vancouver, BC Canada; 3 Centre de Recherche du Centre Hospitalier de I’Université de Montréal, Université de Montréal, Montréal, QC Canada; 4 Department of Biochemistry and Molecular Medicine, Faculty of Medicine, Université de Montréal, Montréal, QC Canada; 5 Laboratory of Molecular Genetics, Institute for Frontier Life and Medical Sciences, Kyoto University, Kyoto, Japan; 6 Laboratory of Molecular and Cellular Immunology, Graduate School of Biostudies, Kyoto University, Kyoto, Japan

**Keywords:** Antimicrobial responses, Microbiology

## Abstract

Host nucleases are implicated in antiviral response through the processing of pathogen-derived nucleic acids. Among many host RNases, decapping enzymes DCP1 and 2, and 5′→3′ exonuclease XRN1, which are components of the RNA decay machinery, have been extensively studied in prokaryotes, plants, and invertebrates but less so in mammalian systems. As a result, the implication of XRN1 and DCPs in viral replication, in particular, the spatio-temporal dynamics during RNA viral infections remains elusive. Here, we highlight that XRN1 and DCPs play a critical role in limiting several groups of RNA viral infections. This antiviral activity was not obvious in wild-type cells but clearly observed in type I interferon (IFN-I)-deficient cells. Mechanistically, infection with RNA viruses induced the enrichment of XRN1 and DCPs in viral replication complexes (vRCs), hence forming distinct cytoplasmic aggregates. These aggregates served as sites for direct interaction between XRN1, DCP1/2, and viral ribonucleoprotein that contains viral RNA (vRNA). Although these XRN1-DCP1/2-vRC-containing foci resemble antiviral stress granules (SGs) or P-body (PB), they did not colocalize with known SG markers and did not correlate with critical PB functions. Furthermore, the presence of 5′ mono- and 5′ triphosphate structures on vRNA was not required for the formation of XRN1-DCP1/2-vRC-containing foci. On the other hand, single-, double-stranded, and higher-ordered vRNA species play a role but are not deterministic for efficient formation of XRN1-DCP1/2 foci and consequent antiviral activity in a manner proportional to RNA length. These results highlight the mechanism behind the antiviral function of XRN1-DCP1/2 in RNA viral infections independent of IFN-I response, protein kinase R and PB function.

## Introduction

Mammalian cells are known to activate intrinsic anti-stress responses when exposed to various environmental stresses, such as viral infection to prolong their survival chance. As part of this stress response, cells temporarily interrupt their translational machinery and shelter mRNAs by sequestering them within cytoplasmic protein complexes, commonly known as stress granules (SGs) [1]. A variety of viruses have been shown to induce the formation of so called antiviral SGs (avSGs), which are important for RIG-I-like receptors (RLRs)-induced type I interferon (IFN-I) responses in cells infected with various viruses, such as Newcastle disease virus (NDV) and encephalomyocarditis virus (EMCV) [2].

In addition to SGs, a subset of cellular proteins involved in cellular RNA degradation colocalize in discrete cytoplasmic domains referred to as processing (P), or glycine-tryptophan (GW) bodies [1]. Components of P-bodies (PBs) include proteins involved in the decapping process such as the DCP1a–DCP2 complex, together with the 5′→3′ exonuclease XRN1 [1]. Moreover, PBs also contain proteins involved in the RNA interference (RNAi) pathway, including Argonaute 1/2 (AGO1/2), and trinucleotide repeat-containing gene 6 A (*TNRC6A*, also known as GW182) [3]. Viruses are affected either positively or negatively by host RNA degradation components. The best studied viruses in this regard are positive-stranded RNA viruses from the *Flaviviridae* family, such as dengue virus, West Nile viruses (WNV), hepatitis C virus (HCV), and yellow fever virus [4–7]. XRN1 acts as an antiviral factor by degrading genomic RNA (gRNA) of flaviviruses. However, the presence of pseudoknot in such viral gRNA limits XRN1 activity, hence resulting in the accumulation of partially digested viral gRNA fragments called subgenomic flavivirus RNA that are toxic to the cells [4–7]. Interestingly, the role of XRN1 and DCPs in viral infections varies greatly. For example, with the help of virus-encoded decapping enzymes, XRN1 has been shown to facilitate efficient replication of *Poxviridae−*Vaccina virus by limiting the accumulation of virus-derived double-stranded RNA (dsRNA) and consequent activation of innate antiviral response [8, 9]. In addition, studies have shown that the presence of XRN1 and DCPs potentially poses a threat to enterovirus, rendering them acquire strategies to destabilize DCPs and XRN1 to prolong viral survival [10].

Despite these previous reports, the broad antiviral role of XRN1 and DCPs during infection of cytoplasmic-replicating RNA viruses remains elusive. Furthermore, there are still significant mechanistic gaps of knowledge about the dynamics and subcellular localization of XRN1 and DCPs, and their interactions with viral nucleic acids as part of the antiviral activity. Here, we analyzed the roles of XRN1 and DCPs in response to cytoplasmic RNA viral infections. We found that infections with RNA viruses from different families, including NDV, EMCV, Sendai virus (SeV), and vesicular stomatitis virus (VSV), trigger the aggregation of XRN1 and DCPs into distinct subcellular compartments containing viral ribonucleoproteins. These aggregates serve as a platform allowing efficient interaction between XRN1/DCPs and viral RNA, consequently inhibiting viral replication. We further demonstrate that XRN1/DCPs optimally exerts its antiviral activity in a manner proportional to vRNA length.

## Results

### XRN1-DCPs drive an antiviral activity independent of IFN-I signaling

**Fig. 1:**
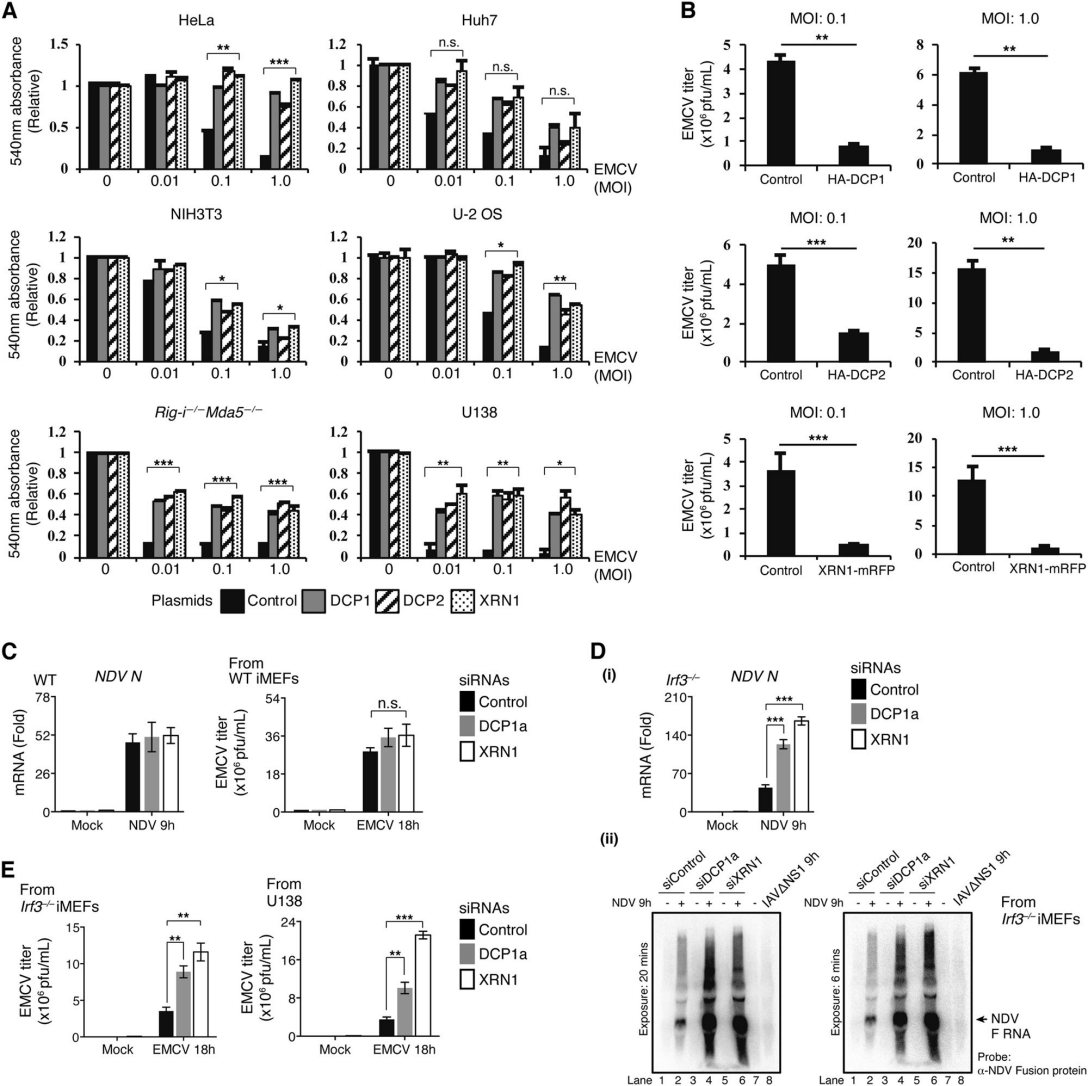
Overexpression of XRN1 and DCP1/2 promotes cell survival by suppressing viral replication. **a** XRN1, DCP1, or DCP2 was ectopically expressed in various cell types for 48 h, followed by infection with indicated MOI of EMCV for 18 h. Crystal violet staining was conducted to determine cell viability. **b** L-929 cells were ectopically expressed with 1.0 µg of indicated plasmids, followed by infection with EMCV at MOI of 0.1 or 1 for 18 h. The released virions were measured by plaque assay. **c** Wild-type (WT) iMEFs were transfected with 1.0 μM of siRNAs for 48 h, followed by infection with NDV or EMCV (MOI = 1) for indicated hours, *NDV N* mRNA was measured by RT-qPCR (left). Supernatant was collected from EMCV-infected cells and viral titers were measured by plaque assay (right); n.s = not significant. **d** *Irf3*^*−/−*^ iMEFs were transfected with indicated siRNAs for 48 h, followed by infection with NDV (MOI = 1) for 9 h. (i) *NDV N* mRNA was measured by RT-qPCR. (ii) *NDV F* (fusion) RNA in each condition was analyzed by northern blot. **e** After EMCV infection (MOI = 1.0) for 18 h, supernatant from siRNA-transfected *Irf3*^*−/−*^ iMEFs and U138 cells was collected. Viral titers were measured by plaque assay. *P*-value in **a** was calculated by two-way analysis of variance, and *P-*value in **b**–**e** was calculated by Student’s unpaired *t*-test.

To characterize the impact of XRN1 and DCP1/2 on cytoplasmic RNA viruses, we first overexpressed DCP1, DCP2, and XRN1 into six different cell lines (HeLa, Huh-7, NIH3T3, U-2 OS, U138, and *Rig-i*^−/−^*Mda5*^−/−^ MEFs). After confirming the expression of these proteins (Supplementary Fig. 1), cells were infected with EMCV. We found that expression of DCP1, DCP2, or XRN1 significantly improved cell survival (Fig. 1a), and reduced virion progeny production (≈5–15-fold at multiplicity of infection (MOI) = 1, Fig. 1b) as compared to control treatment.

To better interrogate the role of XRN1 and DCPs in host antiviral immunity, immortalized murine embryonic fibroblasts (i-MEFs) were transfected with control, XRN1, or DCP1a-specific small interfering RNAs (siRNAs), followed by infection with NDV or EMCV. To our surprise, in contrast to overexpression, knockdown of DCP1a or XRN1 (Supplementary Fig. 2a), exerted minimal effects on both viral RNA levels and titers (Fig. 1c). We speculated that virus-induced IFN-I response masked the antiviral effect of DCPs and XRN1. To test this, we used immortalized MEFs deficient in IRF3 (*Irf3*^*−/−*^ iMEFs), a critical transcription factor for IFN-I-associated gene [11]. In the absence of IRF3, gene silencing of DCP1a or XRN1 (Supplementary Fig. 2b) caused a significant increase in *NDV N mRNA* levels (~ 3–5-fold, Fig. 1d) and EMCV titers (Fig. 1e). These findings were further confirmed in U138 cells, a human brain glioblastoma-derived cell line with the deletion of several key IFN-I genes [12] (Fig. 1e, Supplementary Fig. 2b). Together, these results are consistent with the data using cell lines deficient in cytoplasmic viral RNA (vRNA) sensors RIG-I and MDA5 (*Rig-i*^−/−^*Mda5*^−/−^ MEFs, Fig. 1a), suggesting an antiviral role for XRN1 and DCP1/2 against NDV and EMCV.

### Viral infection triggers redistribution of XRN1 and DCPs to form aggregates within viral replication complexes

**Fig. 2:**
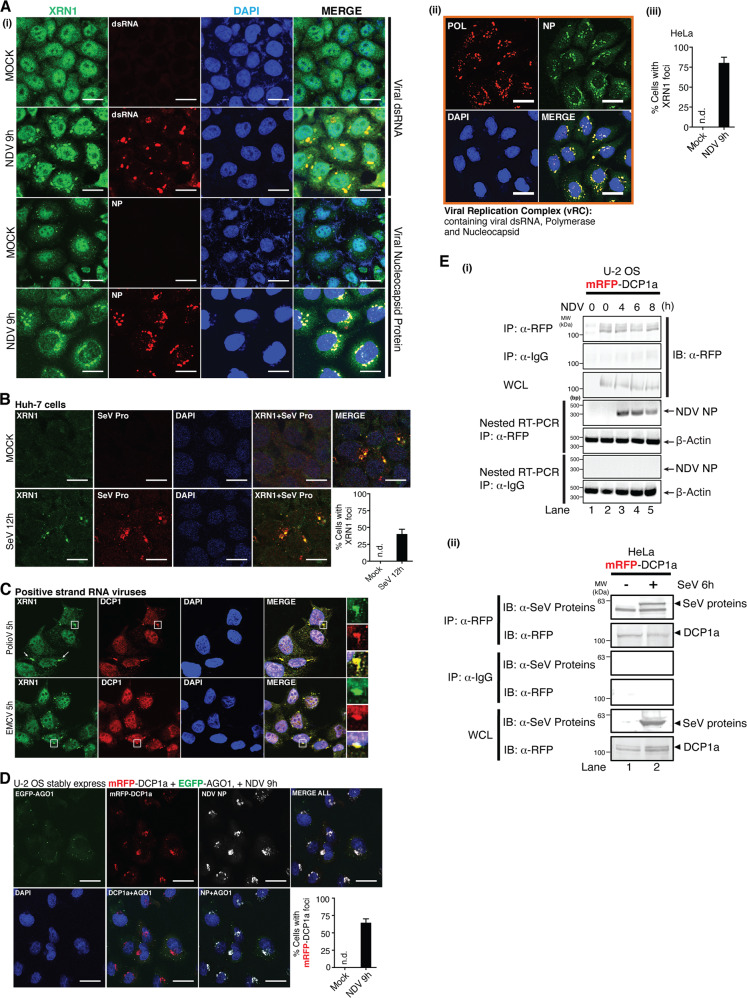
Infection with RNA viruses triggers the translocation of XRN1-DCPs into vRC. **a** (i–ii) Confocal micrographs of XRN1 distribution in NDV-infected cells. HeLa cells were either mock treated or infected with NDV (MOI = 1.0) for 9 h. Immunostaining for endogenous XRN1, viral dsRNA, N proteins (NP), and polymerase (Pol) was conducted. (iii) Percentage of cells with XRN1 foci was quantified. **b** Huh-7 cells were either mock or SeV (MOI = 1.0) infected for 12 h, followed by immunostaining with indicated antibodies. Percentage of cells with foci was quantified. **c** HeLa cells were either mock treated or infected with PolioV or EMCV (MOI = 1.0) for 5 h, followed by immunostaining with indicated antibodies. White box for a specific region was enlarged. **d** U-2 OS cells stably expressing mRFP-DCP1a and EGFP-AGO1 were infected with NDV for 9 h. Immunostaining for viral NP was performed and percentage of cells with Dcp1a foci was quantified. **e** (i) U-2 OS stable cells were either mock treated or NDV infected for indicated time points. Cells were lysed for RNA Co-IP analysis and *NDV N* mRNA was evaluated by RT-qPCR. (ii) Similar IP experiments were performed using HeLa cells expressing mRFP-DCPa, which were infected with SeV for 6 h and immunoblotting was conducted with indicated antibodies. All the white scale bars correspond to 10 μm. n.d., not detected.

To understand the antiviral mechanism of XRN1 and DCPs, we monitored their cellular distribution. The negative strand NDV was used as its viral replication complexes (vRCs) were previously characterized [13]. Microscopy analysis revealed that NDV infection induced a robust aggregation of XRN1 (in ≈75% of cells), colocalizing with vRCs that contain viral dsRNA, viral mRNA, nucleoprotein (NP), and large RNA-dependent RNA polymerase (L) (Fig. 2a, Supplementary Fig. 3a). Infection with another negative-sense virus, SeV was also found to induce the colocalization of XRN1 with vRC (Fig. 2b). As for positive strand viruses, infection with EMCV and poliovirus (PolioV) at early time points, similarly stimulated the aggregation of XRN1 and DCP1a (Fig. 2c). Interestingly, time-resolved immunofluorescence analysis demonstrated that redistribution of DCP1a to vRC took place early, while colocalization of XRN1 with vRC was not observed until 6 h post-NDV infection (Supplementary Fig. 3b). Such redistribution is consistent with the observation of enhanced viral replication in knockdown condition (Supplementary Fig. 3c). Our results suggested a sequential and highly coordinated antiviral response from XRN1 and DCP1a.

To determine whether XRN1-DCPs utilize the RNA-induced silencing complex (RISC) to target vRNA for degradation [14, 15], we used human bone osteosarcoma epithelial (U-2 OS) cells stably co-expressing mRFP-DCP1a and EGFP-AGO1 [16]. Infection of these cells with NDV also triggered an aggregation of mRFP-DCP1a (≥ 60% of cells), which colocalized with NDV NP, but not AGO1 (Fig. 2d), suggesting that AGO1 and its associated RISC pathway were unlikely involved in this event. We further analyzed the relationship between XRN1-DCPs aggregates and vRNA. Figure 2e demonstrated that NDV vRNA and SeV proteins physically interacted with mRFP-DCP1a.

Finally, we asked whether the induced XRN1-DCPs aggregation is driven by IFN-I response. As indicated in Supplementary Fig. 4, IRF3-specific siRNA depletion did not impair virus-induced foci formation. Similarly, activation of IFN-α/β receptor (IFNAR)-pathway by IFN-β did not cause any XRN1-DCPs aggregation, indicating an IFN-I-independent activity.

### Virus-induced XRN1 and DCPs aggregates are discrete foci, but not avSG

Previously, we have shown that avSGs are critical for sensing vRNA during infection by providing a platform with increased local concentration of antiviral effector proteins [17]. To clarify whether the virus-induced XRN1-DCPs aggregates are avSGs, we utilized HeLa cells stably expressing G3BP1 (the avSG marker) fused with EGFP (EGFP-G3BP1) [18, 19]. Following NDV infection, XRN1 aggregated and colocalized with NDV NP, but not with G3BP1 (Supplementary Fig. 5a). HeLa cells stably co-expressing EGFP-G3BP1 and mRFP-DCP1a, and wild-type (WT) HeLa cells immunostained for eIF3η, another SG marker [20], showed similar results (Supplementary Fig. 5b, c). These findings were consistent with the RNA immunoprecipitation (RIP) assay data, exhibiting an association between DCP1a and EMCV RNA, but not G3BP1 (Supplementary Fig. 5d). We also found that formation of G3BP1 foci (avSGs), but not XRN1-DCPs foci, was significantly impaired in protein kinase R (PKR)^KD^ cells compared to control cells (Supplementary Fig. 5e). Together, these results suggested that virus-induced XRN1-DCPs aggregates were not avSGs and their formation was independent of PKR.

### Cytoplasmic-replicating RNA viruses, not DNA viruses, efficiently trigger XRN1-DCPs aggregation

**Fig. 3:**
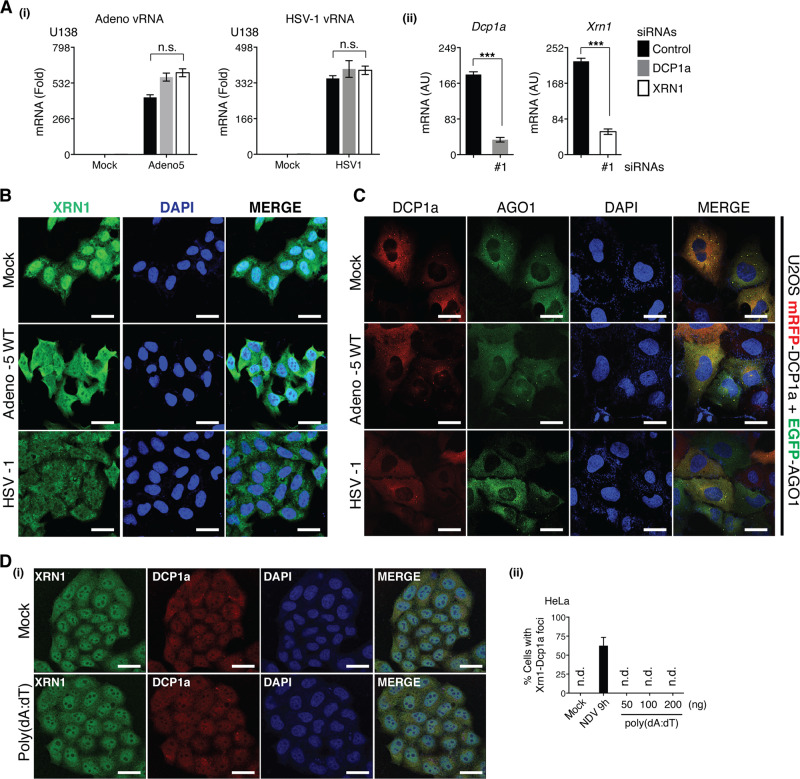
Infection with DNA viruses does not trigger the formation of XRN1-DCPs foci. **a** U138 cells were transfected with 1.0 μM of indicated siRNAs for 48 h, followed by Adeno-5 and HSV-1 (MOI = 1.0) infection for 48 h. (i) vRNA and (ii) knockdown efficiency were measured by RT-qPCR. **b** Both Adeno-5- and HSV-1-infected HeLa cells (MOI = 1.0) were immunostained for endogenous XRN1 (green). **c** U-2 OS cells stably expressing mRFP-DCP1a and EGFP-AGO1 were either mock treated or Adeno-5-, HSV-1 infected for 48 h. Cells were fixed and nuclei were stained with DAPI (blue). **d** (i) HeLa cells transfected with 100 ng of poly(dA:dT) were immunostained for endogenous XRN1 and DCP1a, and (ii) percentage of cells with XRN1-DCP1a foci was quantified. All the white scale bars correspond to 10 μm. *P*-value in **a** (i) was calculated by two-way analysis of variance, and in **a** (**ii**) was calculated by Student’s unpaired *t*-test. AU, arbitrary units; n.d., not detected.

We then asked whether XRN1-DCPs aggregation also occurs during DNA viral infection. Knockdown of DCP1a or XRN1 demonstrated a trend toward increased vRNA accumulation in cells infected with Adenovirus 5 (Adeno-5) and herpes simplex virus-1 (HSV-1). However, these changes were not statistically significant (Fig. 3a). Further analysis revealed that infection with Adeno-5 or HSV-1 failed to stimulate cytoplasmic aggregation of XRN1 and DCP1a (Fig. 3b, c). Transfection of poly(dA:dT), a synthetic analog of B-DNA, produced the same results (Fig. 3d). Overall, our results suggested that the observed XRN1-DCPs redistribution is specific to cytoplasmic RNA viruses, but not nuclear DNA viruses.

### Trimerization domain of DCP1a is critical for XRN1-DCPs-dependent antiviral activity

**Fig. 4:**
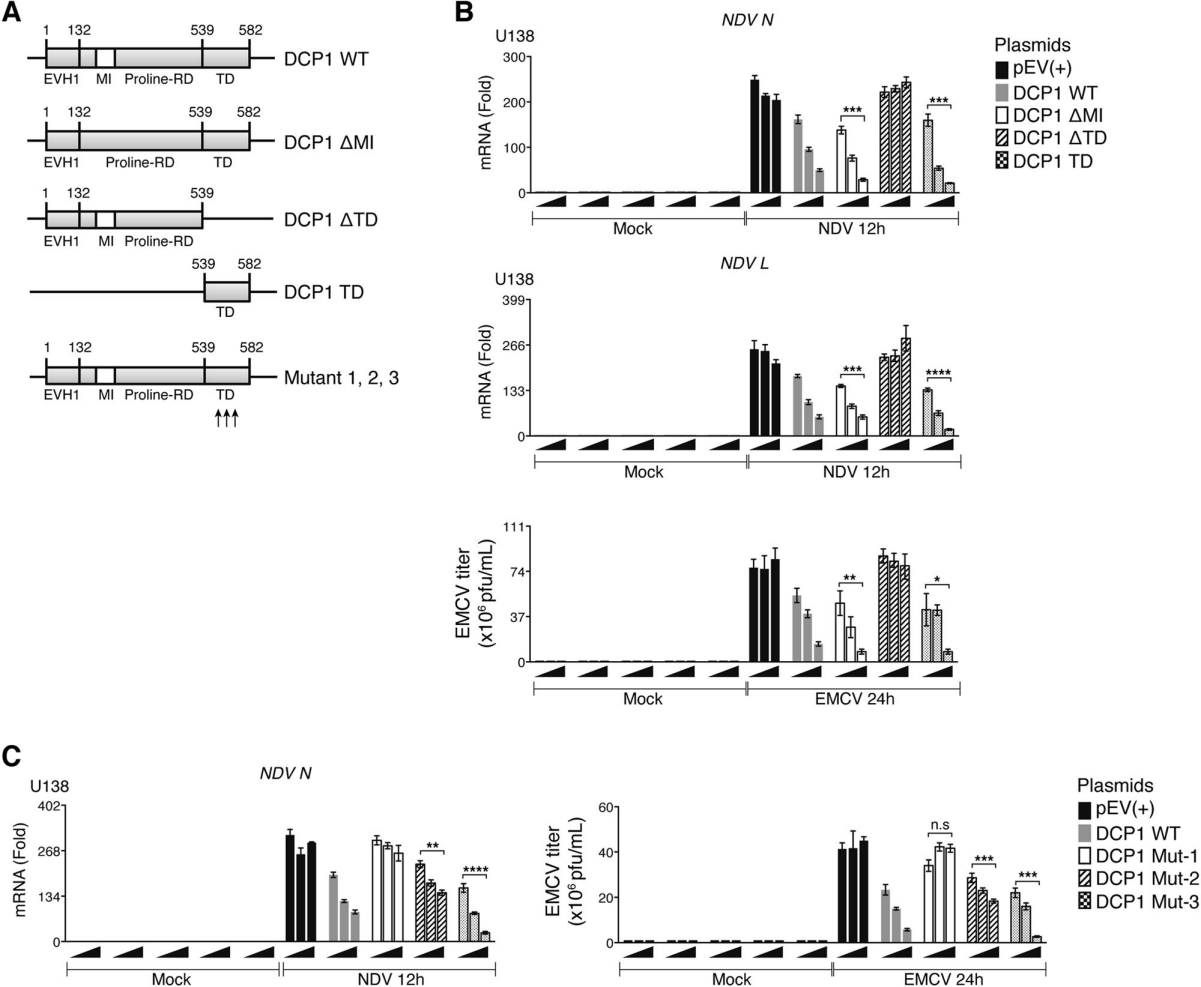
Deletion of the trimerization domain of DCP1 abolishes its antiviral activity. **a** Schematic diagram of human DCP1 deletion and point mutants. **b** U138 cells were ectopically expressed with indicated plasmids at increased dosages of 0.1, 0.5, and 1.0 μg for 48 h, followed by either NDV or EMCV (MOI = 1.0) infection for 12 h and 24 h, respectively. *NDV N* and *L* mRNA was measured by RT-qPCR. Supernatant from EMCV-infected cells was collected for plaque assay. **c** U138 cells were overexpressed with indicated plasmids at the dosages of 0.1, 0.5, and 1.0 μg for 48 h, followed by NDV (MOI = 1.0) infection for 12 h. *NDV N* mRNA was assessed by RT-qPCR. Supernatant from U138 cells infected with EMCV (MOI = 0.1) was subjected to plaque assay. *P*-value was calculated by two-way analysis of variance analysis.

We further characterized the region in DCP1 required for its antiviral activity using vectors expressing deletion mutants lacking the 14-residues short motif I (MI, referred to as DCP1-ΔMI mutant), the trimerization domain (TD, referred to as DCP1-ΔTD mutant), or all domains except for the TD (DCP1-TD mutant, Fig. 4a). Figure 4b showed that mRNA levels of *NDV N* and *L*, and EMCV titers were dose dependently reduced in U138 cells overexpressing WT-DCP1. Deletion of MI domain had no effects on DCP1-driven antiviral activity, whereas TD deletion abolished the antiviral capacity of DCP1 against NDV and EMCV. Of note, overexpression of DCP1-TD alone was able to diminish NDV and EMCV proliferation in U138 cells, suggesting that the TD of DCP1 was crucial for the observed antiviral activity. To further explore underlying mechanism, we partially or completely disrupted the trimerization process by introducing point mutations to the TD. Mutant 1 (L554S, F561R, and L565S) and mutant 2 (I552S, I555R, and L562E) are known to partially lose their interacting capacity between monomers during trimerization, while mutant 3 (L551R, I555S, F561R, and L565S) completely fails to oligomerize [21]. Figure 4c showed that transient expression of mutant 1 abolished the antiviral activity driven by DCP1-WT in NDV-infected U138 cells. In contrast, mutant 2 only weakly attenuated, while mutant 3 had no significant effects on DCP1-dependent antiviral activity. Together, these results suggested that the antiviral activity of DCP1 relied on its TD, but independent of the known function of TD in DCP1 oligomerization.

### Viral single-stranded and dsRNA species, but not viral mRNA, are crucial to trigger the translocation of XRN1-DCPs into vRC

**Fig. 5:**
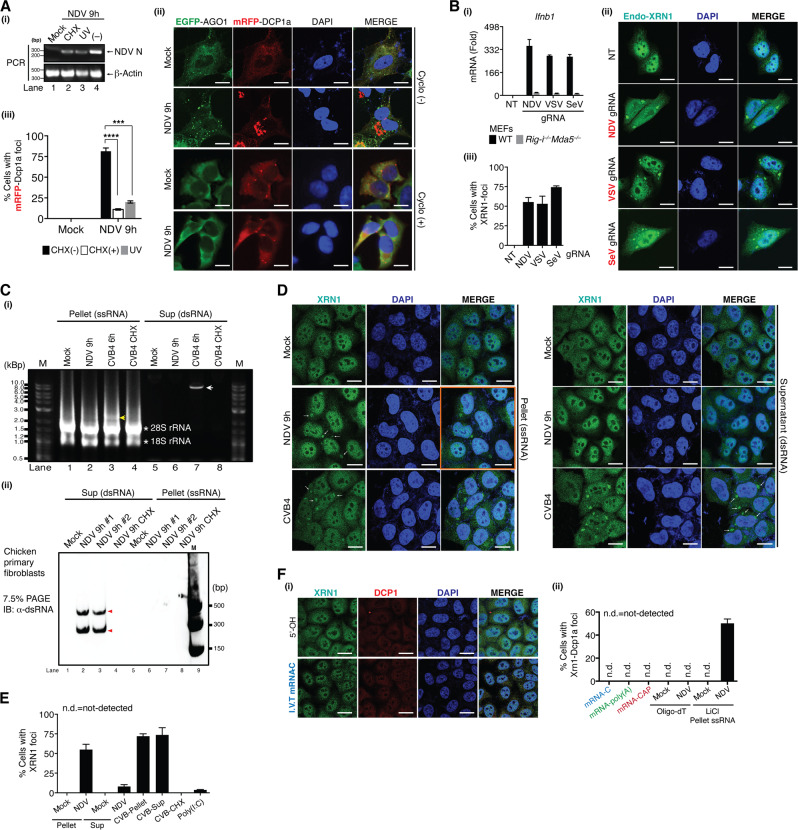
Viral ss- and dsRNA species, but not viral mRNA, are essential to trigger the translocation XRN1-DCPs into vRC. **a** U-2 OS EGFP-AGO1 and mRFP-DCP1a cells were either mock treated or NDV infected (MOI = 1.0) for 1 h. Cells were then exposed to UV (500 millisevert) or CHX (100 μg/mL). Cells were harvested for either (i) RT-qPCR to quantify *NDV N* mRNA, or (ii) immunostaining to visualize DCP1a foci. (iii) Percentage of cells with DCP1a foci was quantified. **b** gRNA from respective viruses was purified, followed by transfection into (i) WT and *Rig-i*^*−*^^*/−*^*Mda5*^*−/−*^ MEFs for *Ifnb1* mRNA measurement by RT-qPCR, or (ii–iii) HeLa cells for immunostaining of endogenous XRN1 and quantitation of percentage of cells with XRN1 foci. **c** CEFs were either mock treated or infected with NDV and CVB4 (MOI = 1.0) for indicated time point. ss- and dsRNA were fractionated and analyzed on (i) agarose gel containing ethidium bromide; (ii) 7.5% PAGE by immunoblotting with anti-dsRNA antibody. **d** Fractionated viral ss- and dsRNA were transfected into HeLa cells and immunostained with indicated antibodies. **e** Percentage of cells with XRN1 aggregates was quantified. **f** (i) I.V.T viral mRNA was transfected into HeLa cells, followed by immunostaining for endogenous XRN1 and DCP1. (ii) Percentage of cells with XRN1-DCP1a foci was quantified. Nuclei were stained with DAPI (blue). All the white scale bars correspond to 10 μm. n.d., not detected.

We next sought to identify viral factors that trigger the redistribution of XRN1-DCPs. U-2 OS cells were treated with cycloheximide (CHX) and ultra-violet (UV) to inhibit viral replication, which was confirmed by significant decreases in viral mRNA (Fig. 5a(i)). Further analysis revealed that treatment with CHX or UV reduced mRFP-DCP1a aggregation (Fig. 5a(ii–iii)), indicating that viral replication intermediates are required for the formation of XRN1-DCPs foci.

To understand the role of vRNA in recruitment of XRN1-DCPs, gRNA of NDV, SeV, and VSV was purified from virions. Consistent with previous studies [22–24], all three gRNAs strongly induced *Ifnb1* expression (≥ 300-fold) in WT-MEFs; and such induction was completely abrogated in *Rig-i*^*−/−*^*Mda5*^*−/−*^ MEFs (Fig. 5b(i)). Furthermore, we showed that introduction of these viral gRNAs was able to trigger the aggregation of XRN1 (Fig. 5b(ii–iii)), suggesting that virion-derived gRNA alone in the absence of active viral replication was sufficient to induce the formation of XRN1 foci.

To narrow down the contribution of specific vRNA species, we fractionated single-stranded (ss)RNA and dsRNA from NDV- and Coxsackie virus B4 (CVB4)-infected cells. Due to discrepancies in replication efficiency, CVB4 produced higher molecular weight of dsRNA compared to NDV (Fig. 5c(i–ii)). We observed that ssRNA species from both viruses, and dsRNA from CVB4 strongly triggered XRN1 aggregation, while synthetic dsRNA, poly(I:C) and NDV-derived dsRNA induced a weaker response (≈5–10% of cells, Fig. 5d, e).

To ask whether viral mRNAs also contribute to XRN1-DCPs aggregation, we synthesized three NDV-derived mRNA species encoding N protein: mRNA with 5′-guanosine cap and 3′-poly(A) tail (referred to as “mRNA-C”); mRNA with only 5′-guanosine cap (referred to as “mRNA-CAP”), and mRNA with only 3′-poly(A) tail (referred to as “mRNA-poly(A)”). Strikingly, none of these mRNA species were capable of triggering the aggregation of XRN1-DCPs (Fig. 5f). Collectively, these results suggested that XRN1-DCPs-vRC foci were induced by cytoplasmic virus-derived ss- and dsRNA species, but not viral mRNA.

### RNA length determines the ability of ssRNA to trigger the formation of XRN1-DCPs foci

**Fig. 6:**
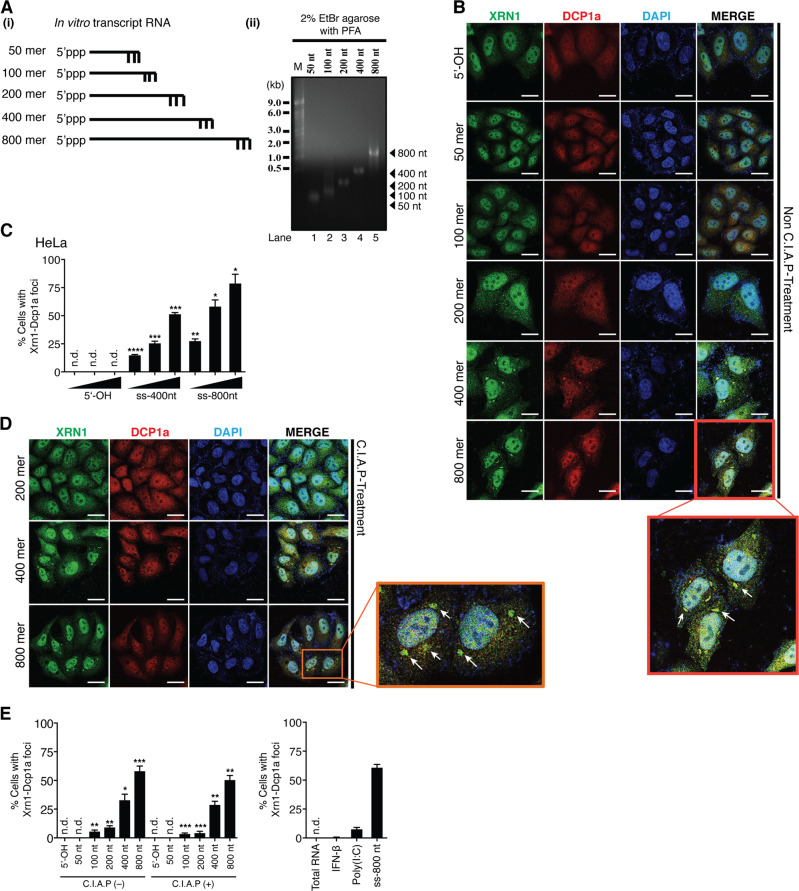
Translocation of XRN1-DCPs into vRC is dependent on RNA length. **a** (i) In vitro transcribed RNA with indicated length was synthesized and (ii) analyzed on 2% agarose gel with EtBr and PFA. **b** Non-C.I.A.P-treated RNA species from **a** were transfected into HeLa cells for 12 h. Immunostaining was performed with indicated antibodies. Nuclei were stained with DAPI. **c** Percentage of cells with XRN1-DCP1a aggregates from **b** for indicated experimental conditions (1.0, 2.5, or 5.0 μg of RNA) was quantified. **d**, **e** C.I.A.P-treated RNA species from **a** were transfected into HeLa cells for 12 h, followed by immunostaining and quantification. Treatments with Poly(I:C) and ss-800 nt RNA were used as positive controls, while treatments with total cellular RNA and 100 U/mL of recombinant IFN-β were used as negative controls. XRN1-DCPs aggregates were indicated by white arrows. n.d., not detected. *P*-value was calculated by Student’s unpaired *t*-test by comparing to control of respective concentrations.

As described above, the dsRNA from CVB4 with higher molecular weight was more efficient in inducing XRN1-DCPs aggregation when compared to the shorter form of dsRNA from NDV. We therefore speculated that the length of vRNA species could be a determining factor. To test this, we generated 5′-triphosphate ssRNA (5′ppp-ssRNA) of various lengths, i.e., 50, 100, 200, 400, and 800 nucleotides (Fig. 6a). We found that only a small fraction of cells (≈13%, refer to Fig. 6e, without calf intestinal alkaline phosphatase treatment) were positive for XRN1 and DCP1a aggregates when 100 nt 5′ppp-ssRNA was transfected (Fig. 6b). However, transfection of 400 nt or 800 nt 5′ppp-ssRNA increased the number of cells positive for XRN1-DCP1 aggregates to ~30% and 50%, respectively (Fig. 6c). In contrast, transfection with either 25 nt 5′-OH or 50 nt 5′ppp-ssRNA failed to initiate foci formation (Fig. 6e), suggesting that the capacity of 5′ppp-ssRNA to trigger foci formation was proportional to RNA length, with a minimum of 100 nt being required. Moreover, transfection with increased amount of 400 nt and 800 nt 5′ppp-ssRNA dose dependently enhanced the number of cells with XRN1-DCP1a aggregates (Fig. 6c), indicating that the intensity of XRN1-DCPs-dependent antiviral activity was also linked to the amount of ss-vRNA species present in the cytosol. Finally, we assessed the relevance of the triphosphate group. As shown in Fig. 6d, e, removal of the triphosphate moiety from 5′ppp-ssRNAs did not alter the ability of ssRNAs to trigger foci formation, suggesting that the 5′-triphoshphate groups play a negligible role in the observed phenotype.

### 5′-monophosphate structure is dispensable for XRN1-DCPs aggregate formation

**Fig. 7:**
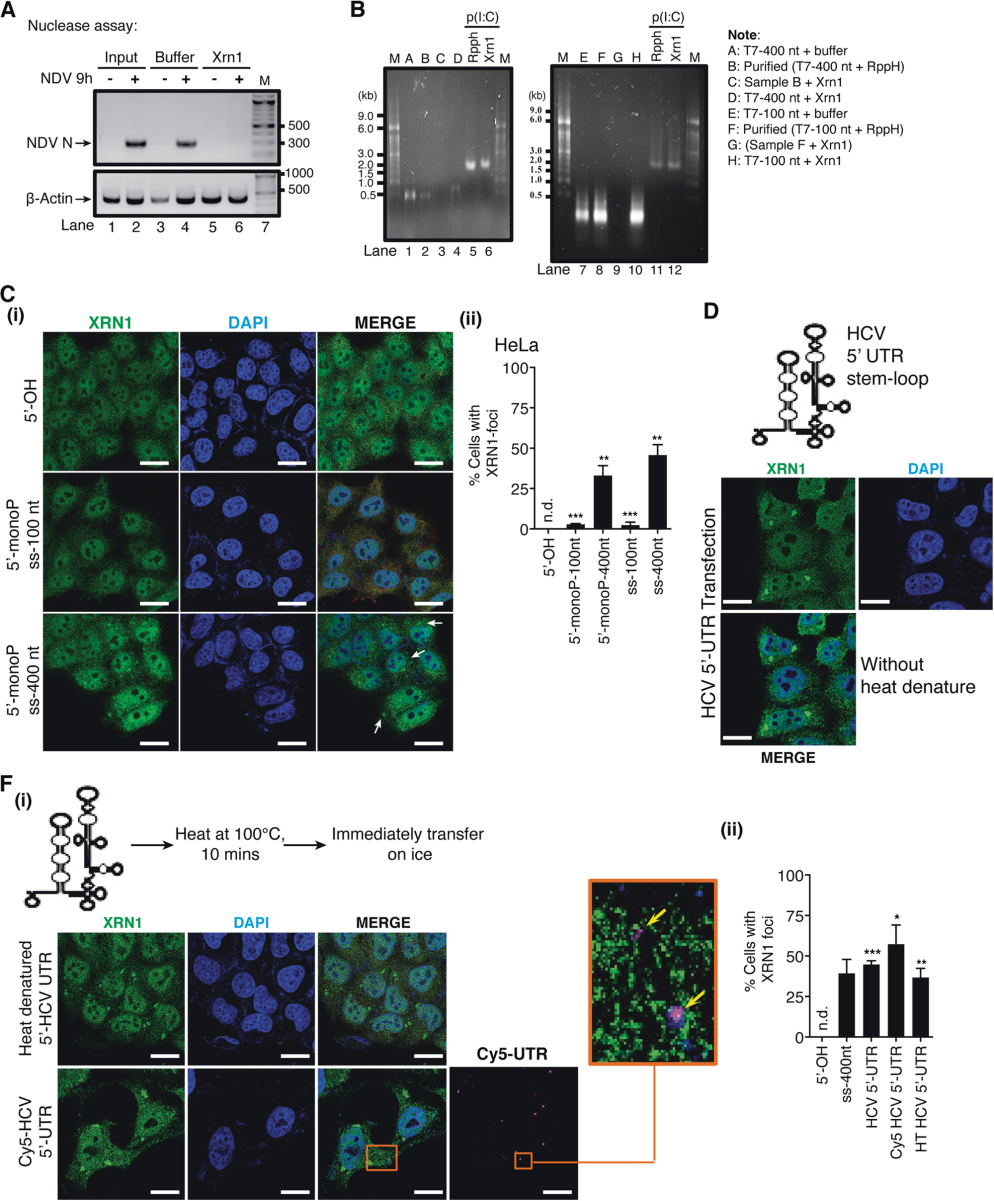
Mono-, tri-phosphate, and multibranch loop structures of vRNAs are dispensable for the formation of XRN1 aggregates. **a** Total RNA extracted from mock- or NDV-infected HeLa cells was treated with or without XRN1 recombinant enzyme. cDNA was then synthesized. *NDV N* mRNA was measured by PCR and analyzed on 2% agarose gel. **b** In vitro synthesized RNA treated under indicated conditions for 12 h was analyzed on agarose gel containing PFA. **c** (i) In vitro synthesized RNA as indicated was transfected into HeLa cells for 12 h. Immunostaining was carried out with XRN1 antibody. (ii) Percentage of cells with XRN1 foci for indicated experimental conditions was quantified. **d** HeLa cells were transfected with (i) intact, or (ii) denatured HCV 5′-UTR RNA, followed by immunostaining with XRN1 antibody. (iii) Percentage of cells with XRN1 foci was quantified. All the white scale bars correspond to 10 μm. n.d., not detected. *P*-value was calculated by Student’s unpaired t-test by comparing to 5′-OH control.

We further assessed whether the vRNA pool contains ssRNA species with 5′-monophosphate group (5′p-ssRNA), a known target for XRN1. Figure 7a (lane 6) showed that vRNA encoding the viral gene *N* was degraded in XRN1-treated samples, suggesting that the 5′p-ssRNA species did exist, rendering them a direct target for XRN1 nucleases.

We then asked whether these 5′p-ssRNA species could induce XRN1-DCPs foci. 5′ppp-ssRNAs were digested with RNA 5′-pyrophosphohydrolase (RppH) to remove pyrophosphates (Fig. 7b, lanes 2 and 8), followed by treatment with recombinant XRN1. No RNA was detected in XRN1-treated samples (Fig. 7b, lanes 3 and 9), indicating successful generation of 5′p-ssRNA and consequent degradation by XRN1. However, no significant differences in XRN1 foci formation were observed in cells transfected with 5′p- or 5′ppp-ssRNAs (Fig. 7c). These results consolidated the findings that neither 5′-guanosine cap, 5′-tri nor monophosphate-bearing RNA species were critical for the formation of XRN1-DCPs foci.

### vRNA multibranch loop structure is not a determining factor for the formation of XRN1 aggregates

A key feature for immune-stimulating capacity is the presence of higher order structures on RNA molecules [25]. We then determined the relevance of higher order structures to XRN1-DCPs aggregation. We found that both native and heat-denatured multiple-loop containing HCV 5′UTR RNA species were able to induce XRN1 foci with efficiency similar to T7-400 nt ssRNA (Fig. 7d). This result suggested that the branch loop structures were not a key factor for XRN1 aggregation.

### PB components are dispensable for virus-induced DCPs redistribution

PB components can differentially regulate viral replication through various mechanisms [26–29]. We next tested the role of critical PB components, including DDX6, Sm-Like homolog 14a (LsM14a), and GW182, in XRN1-DCPs aggregation. Supplementary Fig. 6a, b showed that depletion of DDX6 and GW182 had no major impact on NDV-induced aggregation of mRFP-DCP1a. Similar results were also obtained for LsM14a (Supplementary Fig. 6c), a PB component previously shown to positively regulate RIG-I-dependent IFN-I signaling [30]. Consistent with prior observation, cytoplasmic localization of EGFP-AGO1 remained unchanged. Collectively, these results suggested that PB components are dispensable for the formation of XRN1-DCPs foci.

### Viruses induce XRN1-DCPs aggregation independent of MAVS, TBK1, STING, and NFκB

Upon viral detection, IRF3 and NFκB activation is conferred by the TANK-binding kinase-1 (TBK1), and IKKε protein kinases, which coordinate with mitochondrial activator of virus signaling (MAVS) adaptor, and stimulator of IFN genes (STING) for downstream signaling [2]. We examined the possible impacts of MAVS, TBK1, and STING on XRN1-DCP1/2-mediated antiviral activities and XRN1 aggregation. As shown in Supplementary Fig. 7a–d, a significant enhancement in virus replication upon DCP1a- and XRN1-depletion in MAVS, TBK1, or STING-deficient cells was observed. However, virus-induced XRN1 aggregation was not affected after depletion of MAVS, TBK1, or STING. Protein expression of MAVS, TBK1, STING, and IRF3 remained unchanged upon mock or poly(I:C) transfection. We also examined the possible interplay between XRN1-DCP1/2 and NFκB activation. As shown in Supplementary Fig. 7e, NFκB activities triggered by tumor necrosis factor α (TNFα), lipopolysaccharide (LPS), or poly(I:C) were comparable between XRN1- or DCP1a-depleted and control cells. Consistent with previous report [31], we observed a slight decrease in CVB3 replication after blocking NFκB. Inhibition of NFκB activity through pharmacological inhibitors (i.e., TPCA-1 and BAY 11-7082) did not affect XRN1-DCP1a-mediated antiviral activities and XRN1 aggregation upon infection with CVB3, a positive ssRNA virus (Supplementary Fig. 7f, g). Collectively, these results suggested that MAVS, TBK1, STING, or NFκB were not required to initiate or sustain the relocation of XRN1-DCP1/2 into vRC for the antiviral activity.

### XRN1 and DCP1/2 prevent Bax- and caspase-1-dependent, virus-induced cell death

**Fig. 8:**
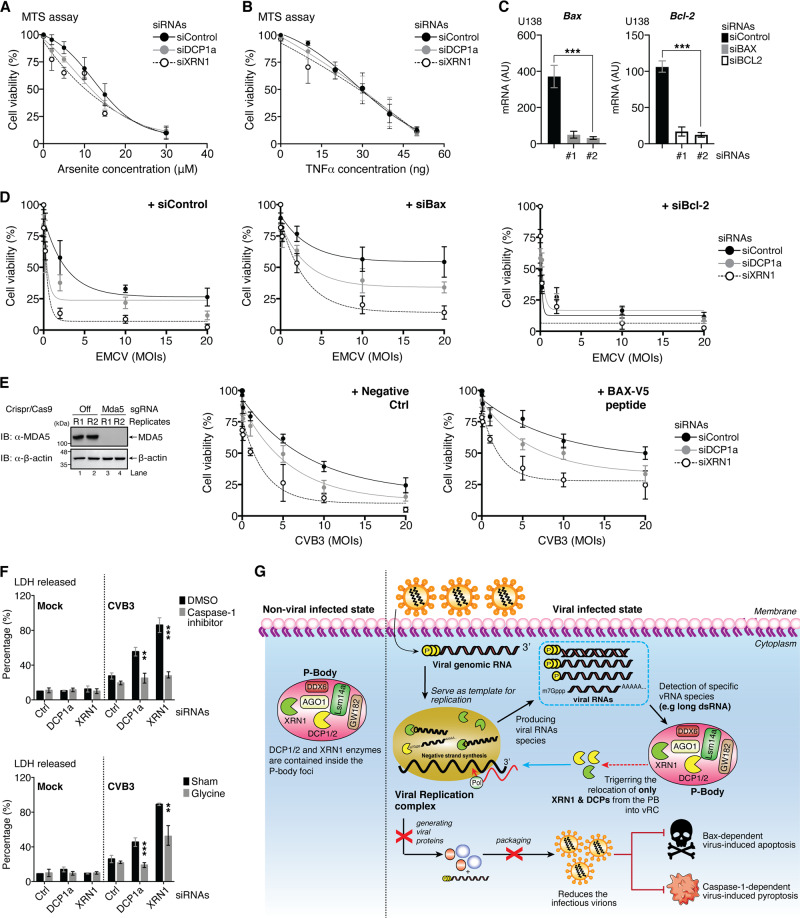
XRN1 and DCP1/2 protect cells from virus-induced, caspase-1-dependent pyroptosis and Bax-dependent apoptosis. **a**, **b** HeLa cells were transfected with indicated siRNAs for 48 h, followed by either arsenite or TNFα treatment at the indicated concentration. Cell viability was measured by MTS assay. **c** Knockdown efficiency of *Bax* and *Bcl-2* gene was measured by qRT-PCR. **d** U138 cells were transfected with indicated siRNAs for 48 h. Cells were then infected with EMCV (MOI = 0, 0.01, 0.1, 2, 10, or 20) for 5 h, followed by MTS assay. **e** MDA5-deficient HeLa cells were generated using *Crispr/Cas9* system. Knockout of MDA5 was confirmed by immunoblotting (left). These cells were transfected with indicated siRNAs for 48 h, followed by treatment with vehicle as negative controls or BIP-V5 peptide (100 μM). Cells were then infected with CVB3 (MOI = 0, 0.01, 0.1, 1, 5, 10, or 20) for 5 h and subjected to MTS assay. **f** MDA5-deficient HeLa cells were transfected with indicated siRNAs for 48 h. Cells were then either treated with dimethyl sulfoxide (DMSO, vehicle), caspase-1 inhibitor (50 **μ**M), or glycine (5 mM), followed by mock or CVB3 infection (MOI = 1) for 5 h. Supernatant was collected to measure LDH release. **g** Schematic diagram indicating that, during quiescent state, decapping enzyme DCP1/2 and host 5′–3′ exonuclease XRN1 are mainly localized in PB. Upon viral infection, viral gRNA released from the virion initiates viral replication process, generating copies of different vRNA species (e.g., ssRNA, dsRNA, and mRNA) in various length or with 5′- or 3′-end modifications. The presence of both ss-vRNA and ds-vRNA with the minimum length of at least 100 nt, or multistem loop structure might trigger the specific relocation (blue arrow) of XRN1 and DCP1/2 decapping enzymes into viral replication complexes for vRNA degradation. Consequently, virus-induced, caspase-1-dependent pyroptosis, and Bax-dependent apoptosis are inhibited. (Statistical analyses in **c**, and **f** are unpaired Student’s *t*-test).

In order to efficiently suppress viral replication, many of the cellular protective measures actually involve the induction of apoptosis and pyroptosis. We then investigated the role of XRN1 and DCPs in these processes. Cell viability assays demonstrated no significant differences in cell survival between DCP1a- or XRN1-depleted cells and control cells after induction of cell death by arsenite or TNFα (Fig. 8a, b), suggesting that XRN1-DCP1/2 play a minimal role in directly regulating cell death pathway.

It is evident that apoptosis induced by Semliki Forest virus requires Bax/Bak [32]. We then examined the impacts of gene silencing of proapoptotic protein Bax and the antiapoptotic protein Bcl-2 on cell viability upon EMCV infection in cells depleted of DCP1a or XRN1 (Fig. 8c). Figure 8d showed that knockdown of Bax attenuated EMCV-induced cell death, whereas depletion of Bcl-2 further enhanced cell death, suggesting a BAX/BCL2-dependent cellular event. To further substantiate this, we used MDA5-deficient cells to prevent potential masking of XRN1-DCP1/2-mediated IFN-I antiviral effect. Similarly, treatment with a Bax-inhibiting peptide markedly inhibited CVB3-induced cell death (Fig. 8e).

To determine the possible involvement of pyroptosis in virus-induced cell death. We treated cells with a caspase-1 inhibitor or with glycine, which maintains the integrity of the cellular membrane [33]. Both approaches were able to attenuate CVB3-induced cell death in DCP1a- or XRN1-depleted cells (Fig. 8f), suggesting that caspase-1-dependent pyroptosis is involved in virus-induced cell death. Together, our findings indicated that XRN1-DCP1/2 complexes could effectively prevent cell death caused by RNA viruses in Bax and caspase-1-dependent manners.

## Discussion

In this study, we demonstrate that cytoplasmic RNA viral infection triggers redistribution of XRN1 and DCPs, leading to the formation of dense aggregates containing viral components, including vRNAs and subsequent inhibition of viral growth. As a result, virus-induced, caspase-1-dependent pyroptosis, and Bax-dependent apoptosis are prevented (Fig. 8g). This event occurs independent of the canonical IFN-I pathway, including STING, MAVS, TBK1, and IFNAR-signaling cascade. Using IFN-I-deficient system, we demonstrate that, depletion of DCP1a and XRN1 significantly enhances both NDV and EMCV replication. Unlike HCV and human immunodeficiency virus that encode viral proteins to counteract IFN-I production [34–38] and depletion of XRN1 increases viral load [28, 29, 39], NDV and EMCV robustly activate the IFN-I pathway and knockdown of XRN1 fails to affect viral replication in cells with intact IFN-I signaling. It remains unclear why IFN-I would mask the XRN1-DCPs-mediated antiviral effect. We speculate that the coordination of several inducible IFN-stimulating genes (ISGs) such as ribonuclease L and 2′5′-oligoadenylate synthetase might compete with cytosolic degradative activity of XRN1-DCPs on vRNA. The resulting RNA fragments can further stimulate the production of IFN-I, amplifying ISG-dependent antiviral activity and resulting in rapid clearance of vRNAs [40, 41].

Notably, we observed that infection with Adeno-5 and HSV-1, two nuclear-replicating DNA viruses, fails to trigger XRN1-DCPs aggregation, and that viral replication remains comparable when DCP1 and XRN1 were depleted in IFN-I-deprived cells. Limited ability of DNA viruses to generate replicative-intermediate RNA species in the cytoplasm may explain their incapacity to induce XRN1-DCPs foci. While we cannot be certain that all DNA viruses would behave similarly, these results provide compelling evidence that nuclear-replicating DNA viruses are less susceptible to this antiviral mechanism.

We have previously demonstrated the importance of avSGs in IFN-I antiviral response [17, 18, 42]. However, we found that the XRN1-DCPs-containing foci are not SGs as they do not colocalize with well-known SGs markers (G3BP1 and eIF3η) and DCP1a does not precipitate with G3BP1. Although both XRN1 and DCPs are well-established PB markers, depletion of GW182 and DDX6, known to disrupt PB architecture [43], does not impair XRN1-DCPs aggregation. In addition, localization of AGO1, another PB marker, remains unchanged upon viral infection, indicating that redistribution of XRN1-DCPs into vRC is independent of PB-related functions. Nevertheless, the possibility that the XRN1-DCPs-vRC specific foci and avSGs components are dynamically shuttling from one compartment to another cannot be excluded [44].

In this study, we demonstrate that the TD of DCP1 is critical for XRN1-DCPs-dependent antiviral activity. Trimerization via TD has been reported to be required for incorporation of DCP1 into the XRN1-DCPs complex by binding to scaffold protein, enhancer of mRNA-decapping protein 4 [45], Intriguingly, we found that transfection of TD alone is sufficient to drive the observed antiviral activity of DCP, while the TD-deleted DCP1 fails to induce antiviral activity. Further analysis revealed that mutant 3 known to abolish trimerization has no effect on XRN1-DCPs-dependent activity, indicating a trimerization-independent mechanism. However, mutant 1 (partially affects trimerization) completely abolishes the observed phenotype, suggesting that the residue L554 could be a key amino acid for the observed antiviral activity. Although exact mechanism by which TD of DCP1 exerts its antiviral function is unclear, we speculate that TD alone could engage DCP2 and XRN1 in a manner dependent on L554 residue and the decapping enzymatic activity of DCP1 is not absolutely required for the antiviral activity of XRN1-DCPs.

We also provide mechanistic insight by showing that XRN1-DCPs-dependent antiviral activity is triggered upon the availability of specific vRNA species within cytosol. XRN1 is known to specifically target 5′p-RNA. Consistent with a previous report [46], we confirm the presence of 5′p-ssRNA species in the cytosol. We demonstrate that 5′p-ssRNA and 5′ppp-ssRNA of optimal length (400 nt) have equal capability of inducing XRN1-DCPs foci formation. Our result further reveals that multiple stem loop structures of vRNA do not appear to be essential for the formation of XRN1 aggregates. The identification of nascent ss- and ds-vRNA species with optimal length as an initiator of XRN1-DCPs-dependent antiviral activity supports the existing concept whereby initiation of a host defense requires the detection of distinct viral signatures. This suggests the possibility of the existence of a cytoplasmic sensor upstream of XRN1-DCPs. Nevertheless, constant interaction between XRN1-DCPs with various RNA binding proteins (RBPs), including heterogenous nuclear ribonucleoprotein K, LSm, DDX, for an optimal degradative function during RNA processing are well established [27, 47, 48]. Many of these RBPs are functionally involved in either positively or negatively regulating viral replication. For instance, GW182, LSM1, DDX3, and DDX6 are shown to be colocalized with XRN1 in WNV replication complex [49]. Although we have excluded some of these components, we cannot completely omit the possibility that recruitment of XRN1-DCPs into vRC could occur indirectly through RBP. The mechanism by which vRNA length determines the intensity of XRN1-DCPs-dependent antiviral activity remains to be fully defined. At least two possibilities exist. Firstly, longer ss- and ds-vRNAs possess higher affinity to fold into complex structures (also known as viral RNA-web) [50]. These rigid structures would require a higher local concentration of exonucleases to achieve complete degradation. Alternatively, longer ss- and ds-vRNA possess additional nucleotide advantage when compared to shorter form, providing additional space to bind with more RBP:XRN1-DCPs complexes, resulting in the observed dense aggregation of XRN1-DCPs.

In summary, our study demonstrates that mammalian DCPs and XRN1 inhibit cytoplasmic RNA virus replication inside a discrete cytoplasmic domain, a granule-like foci independent of SGs and PBs, by directly interacting with vRC. We also narrow down on the identity of vRNA species that can efficiently trigger the redistribution of XRN1-DCPs complexes into these compartments. These findings advance our knowledge on how cells control the amount of ligands capable of activating RLRs, hence preventing an exaggerated IFN-I response that could be detrimental to the host. As viruses possess strategies to bypass IFN-I response, IFN-I-based antiviral therapeutics could be very challenging. Our study hereby also shows that function of XRN1 and DCPs could be tightly regulated to specifically target vRC apart from host mRNA molecules. This provides an additional possibility that these host factors could be exploited as a novel IFN-independent avenue for improved antiviral treatments with minimal harm to the host.

## Materials and methods

### Cell lines, cell culture, and transfection conditions

HEK293T (#CRL-3216, ATCC), Huh-7 [51, 52], HEp-2 (#CCL-23, ATCC), U-2 OS (#HTB-96, ATCC), U138 (#HTB-16, ATCC), NIH3T3 (#CRL-1658, ATCC), HeLa cells (#CCL-2.2, ATCC), L-929 (Strain L, #CCL-1, ATCC), chicken embryonic fibroblasts (CEF, isolated from 9-days old specific-pathogen-free embryonated chicken eggs, Charles River #10100332) [53], and all the murine fibroblasts related cells (isolated from embryos under SPF C57BL/6 background mice, Japan SLC, Inc.) were maintained in Dulbecco’s modified Eagle’s medium (DMEM; Nacalai Tesque) containing 10% fetal bovine serum (FBS). L-929 cells were maintained in minimal essential medium (Nacalai Tesque) containing 5% FBS. DNA-related materials were transfected with Lipofectamine2000 (Invitrogen) according to manufacturer’s protocol. RNA-related materials were transfected with RNAi-MAX (Invitrogen) by manufacturer’s protocol. The U-2 OS cells stably expressing mRFP-DCP1a and EGFP-AGO1 were kindly provided by Nancy Kedersha (Brigham and Women’s Hospital, Boston, USA). HeLa sh-Control and sh-PKR cells were kindly provided by Charles E. Samuel (UCSB, California, USA) [54]. The extraction of fibroblasts cells from both SPF mice and embryonated chicken eggs was approved by the Animal Research Committee of Kyoto University of Japan.

### Plasmid constructs

Human HA-DCP1, HA-DCP2, and other DCP1 mutant constructs were kindly provided by Elisa Izaurralde (Max Planck Institute, Tübingen, Germany). Human mRFP-DCP1a and EGFP-AGO1 constructs were kindly provided by Nancy Kedersha (Brigham and Women’s Hospital, Boston, USA) [16]. Both the human and murine XRN1 cDNA were kindly provided by John L. Goodier [55], and were cloned into different plasmid backbone following standard cloning procedures.

### Virus and infection

NDV (strain Miyadera/51) and SeV (Cantell) were propagated in chicken eggs. Briefly, the virus was inoculated into 9-day embryonated chicken eggs and incubated for 2 days at 37 °C, followed by overnight incubation at 4 °C. Allantoic fluid containing the propagated virus was collected. EMCV (Rueckert strain), VSV (Indiana strain, M mutant), CVB3 (Nancy strain), CVB4, HSV-1, and Adeno-5 were propagated by infecting BHK21 cells at a MOI of 1.0. Cell culture medium was collected after confirming the cytopathic effects following infection. Medium containing newly produced viruses was centrifuged at 1,500 rpm for 5 min to pellet the cell debris, and supernatants containing viruses were collected and stored at −80 °C. For infection, in brief, virus was added to cell at a MOI of 1.0. After 1 h incubation at 37 °C, the medium was replaced with fresh media and incubated for the indicated hours of infection.

### vRNA purification

For viral gRNA purification, the allantoic fluid derived from the virus-inoculated chicken eggs was collected and centrifuged at the maximum speed overnight. The supernatant was discarded and TRIzol solution was added to the pellet. RNA extraction and purification were performed according to manufacturer’s instruction. ssRNA and dsRNA fractionation from mock and viral-infected cells was performed as previously described [56].

### Recombinant proteins, agonists, and pharmacological inhibitors

Recombinant human TNFα (#RC214-12) was from Bio Basic; NFκB inhibitor BAY 11-7082 (#S2913) was from Selleck Chemicals; Caspase-1 inhibitor VX-765 (#inh-vx765i-1), and high molecular weight poly(I:C) (#tlrl-pic) was from Invivogen; TPCA-1 (#T1452), LPS (#L5024), and glycine (#67419) were all from Sigma.

### Immunoblotting and antibodies

Cells were harvested in ice-cold phosphate-buffered saline (PBS) using a cell scraper. Cells were pelleted down by centrifugation and lysed in NP-40 lysis buffer (50 mM Tris [pH 8.0], 150 mM NaCl, 1% [vol/vol] NP-40, 1 nM vanadate, 1 mM leupeptin, and phenylmethanesulfonyl fluoride), followed by centrifugation at maximum speed for 10 min and ultracentrifugation at 100,000 rpm for 5 min. The supernatant containing whole cell extract was mixed with an equal volume of 2× sodium dodecyl sulfate (SDS) buffer, boiled at 100 °C for 5 min before being immediately transferred on ice and incubated for 5 min. The sample volume corresponding to a protein amount of 30 μg was applied through a 5–20% gradient e-PAGEL (ATTO) using standard SDS–polyacrylamide gel electrophoresis (PAGE) protocol before being transferred onto an Immobilon-P PVDF membrane (MILLIPORE). The membranes were then incubated in blocking buffer (PBS, 5% [wt/vol] dry milk powder) for 30 min at room temperature (RT), followed by incubation with primary antibody diluted in blocking buffer at 4 °C overnight. Membranes were washed extensively with TBST (Tris-buffered saline, 0.04% Tween 20), followed by incubation with an AP- or HRP-conjugated secondary antibody for 1 h at RT. After washing with TBST, protein bands were visualized using the BCIP-NBT Solution Kit for Alkaline Phosphate Stain (Nacalai Tesque) or ECL Prime Western Blotting Detection Reagent (GE Healthcare). The primary antibodies used in these study were: anti-DCP1a (#sc-22575, Santa Cruz Biotechnology), anti-XRN1 (H-150 #sc-98459, Santa Cruz Biotechnology), the anti-NDV NP mouse monoclonal antibody produced by Dr. Y. Nagai, and provided by Dr. T. Sakaguchi (Hiroshima University, Japan), anti-SeV (MBL, #PD029), anti-HA (#H9658, Sigma-Aldrich), anti-dsRNA/J2 (Scicons), anti-RFP (#ab62341, Abcam), anti-G3BP1 (#sc-365338, Santa Cruz Biotechnology), anti-eIF3η (#sc-16377, Santa Cruz Biotechnology), and anti-β-Actin (#A2228, Sigma-Aldrich). Anti-MDA5 (D74E4), anti-MAVS (D5A9E), anti-STING (D2P2F), anti-TBK1 (D1B4), and anti-IRF3 (D6I4C) were all from Cell Signaling Technology. NDV-L antibodies were originally generated by immunizing rabbits with synthetic peptides targeting amino acids 1160–1183 of NDV-L. The secondary antibodies used were: goat anti-rabbit IgG-AP (#sc-2007, Santa Cruz Biotechnology), goat anti-mouse IgG-AP (#sc-2008, Santa Cruz Biotechnology), anti-rabbit IgG, HRP-linked (#7074, Cell Signaling Technology), and anti-mouse IgG, HRP-linked (#7076, Cell Signaling Technology).

### Northern blotting, protein, Co-IP and RIP assay

Immunoprecipitation was performed using total protein extracts from respective cells in various conditions (~300 mg), together with 1 mg of respective antibodies (as indicated in the figures). After overnight incubation at 4 °C, immune complexes were precipitated together with protein A-Sepharose beads (Amersham Biosciences) and analyzed by immunoblotting as described above. RIP assay was performed using total protein extracts from mock- or viral-infected cells with indicated antibody by RiboCluster Profiler RIP-Assay Kit (MBL, Japan, RN1001) according to the manufacturer’s recommendations. In brief, RNA–protein complex was pulled down either with 5 mg of mouse normal IgG (#sc-2025, Santa Cruz Biotechnology) or antibodies of interest. The bound RNAs were then recovered from the RNA–protein complex and used as a template for cDNA synthesis. Reverse-transcription and quantitative-PCR (RT-qPCR) was further performed to evaluate the RNA level bound to respective pulled-down proteins with the specific primer sets targeting the respective viral-associated gene. As an internal control, the glyceraldehyde 3-phosphatedehydrogenase (GAPDH) gene was used. Northern blotting was performed as previously described [13]. RNA probes to detect NDV F-mRNA was prepared from NDV cDNA using primer sets: Forward 5′-GCACAGATAACAGCAGCCTC-3′ and reverse 5′-CATCTTCCCAACTGCCACTG-3′.

### RNA preparation and RT-qPCR

RNA was harvested from cells using TRIzol (Invitrogen) according to the manufacturer’s instructions. Genomic DNA contamination was removed by treating the RNA extracts with recombinant DNase I (10 units/μL; Roche) at 37 °C for 1 h. Treated samples were purified through phenol-chloroform extraction. Total of 500 ng of purified RNA was used as a template to synthesize cDNA using a High Capacity cDNA reverse transcription kit (Applied Biosystems), as recommended by the manufacturer. The concentration of newly synthesized cDNA was quantified using spectrophotometer, and the final concentration adjusted to 1.0 μg/μL. cDNA samples were then subjected to gene expression analysis using either standard PCR or RT-qPCR analysis with specific TaqMan probes (Applied Biosystems). Quantification of NDV N mRNA was performed using SYBR master mix reagent (Applied Biosystems, CA, USA, #4385612) with specific primers targeting the NDV N protein coding region. All gene expression analyses were then processed through the 2^−ΔΔCt^ relative quantitative method. Primer sequences for measuring EMCV capsid-coding region, NDV N, HSV-1, and adeno-5 mRNA have been previously described [13, 18, 57, 58]. Sequence information (both *Homo sapiens* and *Mus musculus* species) for validated primers for other genes sets (e.g., *Dcp1a*, *Xrn1*, *Trex1*, *Bcl-2*, *Bax*, *Mavs, Sting*, *Tbk1*, *Gapdh*, and *Lsm14a*) can be obtained from Primer-Bank repository site. (https://pga.mgh.harvard.edu/primerbank/). Primers were all customized and purchased from Invitrogen.

### RNA Interference

The siRNAs targeting DCP1a (Human: HSS125069, HSS125070, HSS183281; Murine: MSS178576, MSS178577, MSS178578), XRN1 (Murine: MSS280619, MSS280620, MSS280621; Human: HSS122909, HSS182510, HSS182511), DDX6/RCK/p54 (HSS102718, H22102719, HSS102720), LsM14a (HSS119772, HSS178043, HSS178044), GW182/TNRC6A (#S26154), IRF3 (HSS105505, HSS105506, HSS105507), and a universal negative control (#12935–300), were purchased from Life Technologies, MDA5 siRNA (Human, sc-61010) was from Santa Cruz. siRNAs against BAX (Human: S1888 and S1889), BCL2 (Human: #214532 and #214533), STING/TMEM173 (Human: HSS139156 and HSS139157), MAVS (Human: HSS148537 and HSS148538), and TBK1 (Human: #899 and #900) were from Thermo Fisher. These siRNAs were transfected at a final concentration of 1.0 μM using Lipofectamine RNAi-MAX Reagents (Life Technologies) according to the manufacturer’s recommendations. At 24 h after transfection, the cells were divided into an equal ratio and then transferred to new culture plates with fresh DMEM. At 48 h post-transfection, cells were harvested or infected with viruses for further experiments.

### Immunofluorescence microscopy analysis and RNA labeling

For immunofluorescence analysis, cells were seeded into an eight-well chamber slide and incubated at 37 °C. After overnight incubation, cells were subjected to various treatment as indicated in respective figures, then washed with sterile PBS for several times, fixed with 4% paraformaldehyde (PFA) solution for 10 min at RT, washed with PBS for two additional rounds, permeabilized with acetone–methanol (mixed in ratio 1:1) for 1 min. Blocking step was perfomed using PBS containing 0.04% Tween 20 (PBST) and bovine serum albumin (5.0 mg/mL) for 1 h at 4 °C. Cell were then incubated with respective primary antibodies overnight followed by fluorophore-conjugated secondary antibodies (Invitrogen). Cells were then washed with PBST extensively and mounted. Oligonucleotides labeling with Cy5-dye was performed following the manufacturer’s instructions (Turbo Labeling Kit, Thermo Fisher, KIT0610). All images were obtained using a Leica CTR 6500 instrument. The secondary antibodies used were: Alexa Fluor 488 donkey anti-rabbit IgG H + L (#A-21206), Alexa Fluor 488 donkey anti-mouse IgG H + L (#A-21202), Alexa Fluor 488 Donkey anti-Goat IgG H + L (#A-11055), Alexa Fluor 594 Donkey anti-Rabbit IgG H + L (#A-21207), Alexa Fluor 594 Donkey anti-Mouse IgG H + L (#A-21203), Alexa Fluor 633 Goat anti-Mouse IgG H + L (#A-21050), Alexa Fluor 633 Donkey anti-Goat IgG H + L (#A-21082), and Alexa Fluor 647 Donkey anti-Goat IgG H + L (#A-21447); all were purchased from Life Technologies. RNA-Fluorescence *In Situ* Hybridization (RNA-F.I.S.H) assay was performed as previously described [13], using the similar kits and RNA probes set.

### In vitro transcription, phosphorylation, and nuclease assay

In vitro transcription was performed as previously described [59]. In brief, 5′ppp-ssRNA was synthesized by in vitro transcription using the T7 Megascript kit (Ambion, Austin, TX) through a template that is chemically synthesized from Japan Bio Services Co., Ltd. (Saitama, Japan). As indicated, oligonucleotides were 5′-phosphorylated, or dephosphorylation was performed by T4 polynucleotide kinase or alkaline phosphatase (TaKaRa), respectively. The NDV mature mRNA was in vitro synthesized as previously described [13] using T7 mScript Standard mRNA kit (CellScript). The 5′-monophosphate RNA was generated using RppH (NEB #M0356S) according to manufacturer’s recommendation. For XRN1 nuclease assay, both in vitro synthesized and cell-purified RNA were subjected to digestion by recombinant XRN1 protein (NEB, #M0338S) according to manufacturer’s protocol.

### Lentiviral vectors and generation of MDA5 CRISPR/Cas9-deficient cells

Lentiviral vectors targeting human MDA5 (*Ifih1* gene) single guide RNAs (sgRNAs) were obtained from Applied Biological Materials. HEK293T cells were transfected with pRSV-Rev, pMDLg, pVSV-G plasmids (Addgene), and the pLenti-IFIH1 sgRNA using Lipofectamine 2000. Supernatants containing lentiviral particles were collected after 48 h and concentrated through Amicon Ultra-15 filter (100 K, Millipore-Sigma). HeLa cells were then transduced with the lentiviral vectors by directly adding concentrated supernatant together with polybrene (5.0 μg/mL, Millipore-Sigma) to cells. Polyclonal cells with positive transduction were selected through puromycin (2.0 μg/mL).

### Cell viability and luciferase assay

3-(4,5-dimethylthiazol-2-yl)-5-(3-carboxylmethoxyphenyl)-2-(4-sulfophenyl)-2H-tetrazolium salt (MTS), and lactate dehydrogenase (LDH) release assays were performed using a CellTiter 96 Aqueous Non-Radioactive Cell Proliferation Assay kit (Promega) and a CyQUANT^TM^ LDH cytotoxicity assay (Invitrogen), respectively, based on manufacturers’ instructions. The luciferase assay was performed with a dual luciferase reporter system (Promega). As the internal control, pRL-TK (Promega) was used.

### Plaque assay and crystal violet assay

Both siRNA negative control and siRNA target-treated cells, as indicated in the respective figures, were infected with EMCV for 24 h. The culture supernatant was collected and centrifuged at maximum speed for 1 min at RT. The supernatant was then collected, and virus yield in the culture supernatants was determined by plaque assay on HEp-2 cells as previously described [60]. As for the crystal violet cell viability staining, in brief, cell culture medium from mock and infected cells was aspirated before washed with sterile PBS, followed by incubation with 0.5% crystal violet solution for 30 min (on a shaking platform at RT). Cell monolayer was then rinsed with tap water several times. After washing, plates were inverted and with mild tapping the residual water was removed. Then plates were air-dried at RT. Finally, methanol was added into each well and incubated on a shaking plateform at RT for 20 min, the optical density at 540 nm wavelength (OD_540nm_) was measured.

### Quantification of cells with virus-induced XRN1-DCP1 aggregates

Quantification was performed as described previously [18]. In brief, for fixed cells, 100 pictures were taken randomly at ten different locations within one well of the eight-well glass chamber. Cells displaying foci were quantified manually in each picture for each location.

### Statistical analysis

All statistical analyses in this study were performed using GraphPad Prism. Statistical significances were indicated as *P < 0.05, **P < 0.01, and ***P < 0.001. Immunoblotting, co-immunoprecipitation (Co-IP), and immunofluorescence experiments are representative of three biological replicates (*N* = 3). Gene expression and aggregates quantitation are presented as the mean of four biological replicates (*N* = 4, mean ± s.e.m.).

## Supplementary information

Figure legends for supplemental materials

Supplemental Figure 1

Supplemental Figure 2

Supplemental Figure 3

Supplemental Figure 4

Supplemental Figure 5

Supplemental Figure 6

Supplemental Figure 7

## Data Availability

All relevant data are within the manuscript and it’s supporting Information files.
