# Supplementary material for: Spatio-temporal characterization of the antiviral activity of the XRN1-DCP1/2 aggregation against cytoplasmic RNA viruses to prevent cell death
Source: Cell Death Differ. 2020 Feb 7;27(8):2363–82. doi: 10.1038/s41418-020-0509-0 (PMC7370233; doi:10.1038/s41418-020-0509-0)
Supplement: Supplementary file 1 — Figure legends for supplemental materials [file 41418_2020_509_MOESM1_ESM.docx]

**Supplementary Information**

**Supplemental Figure 1. Protein expression upon HA-DCP1/2 overexpression.**

1.0µg of HA-DCP1/2 plasmids were ectopically-expressed in various cell-types. The expression of HA-DCP1/2 was analyzed through immunoblotting.

**Supplementary Figure 2. Knockdown efficiency of DCP1a and XRN1 siRNAs.**

(**A**-**B**) U138, WT and IRF3-deficient MEFs were transfected with 1.0µM of indicated siRNAs for 48h. Knockdown efficiency for each component was measured by RT-qPCR. *P*-value was calculated by Student’s unpaired *t*-test.

**Supplementary Figure 3. DCP1a and XRN1 sequentially target vRC**

(**A**) NDV-infected HeLa cells were immunostained with indicated antibodies. NDV mRNA was detected by RNA-F.I.S.H method. (**B**) Confocal images of mock or NDV-infected HeLa cells that were immunostained with indicated antibodies. (**C**) HeLa cells were transfected with indicated siRNAs for 48h, followed by NDV infection at indicated time-points. *NDV N* mRNA was quantified by RT-qPCR. All the white scale bars correspond to 10μm. *P*-value in panel **C** was calculated by two-way ANOVA.

**Supplementary Figure 4. Virus-induced redistribution of XRN1 and DCP1a is independent of type I IFN signaling.**

(**A**) (i) HeLa cells were transfected with IRF3 siRNAs and knockdown efficiency was quantified by RT-qPCR, (ii) Following the similar immunofluorescence assay and percentage of cells with XRN1-DCP1a foci was quantified. (iii) *Ifnb1* gene expression in HeLa cells treated as indicated was determined by RT-qPCR. (**B**) (i) Confocal images of mock or (100 U/mL) IFNβ-treated HeLa cells. (ii) Percentage of cells with XRN1-DCP1a foci for respective experimental condition was quantified. *P*-value in panel **A** was calculated by Student’s unpaired *t*-test. All the white scale bars correspond to 10μm. n.d. = not detected.

**Supplementary Figure 5. Virus-induced XRN-DCP1a complexes do not contain SG components.**

(**A**) Confocal images of NDV-infected HeLa cells stably expressing EGFP-G3BP1, which were immunostained for NDV NP and endogenous XRN1. (**B**) Confocal images of mock or NDV-infected HeLa cells stably co-expressing EGFP-G3BP1 and mRFP-DCP1a, which were immunostained with NDV NP antibody. (**C**) HeLa cells were either mock or NDV-infected, followed by immunostaining for eIF3η and NDV NP. (**D**) HeLa-mRFP-DCP1a stable cells were either mock-treated or EMCV-infected for 5h. Cells were lysed for RNA Co-IP analysis, and EMCV capsid coding mRNA was evaluated by RT-qPCR. (**E**) Similar immunofluorescence staining was performed using mock- or NDV-infected HeLa cells transfected with sh-Control or sh-PKR. Percentage of cells with XRN1 or G3BP1 foci was quantified. All the white scale bars correspond to 10μm. n.d. = not detected.

**Supplementary Figure 6. Virus-induced redistribution of XRN1 and DCP1a does not require critical PB components.**

(**A**) U-2 OS cells were transfected with indicated IRF3 siRNAs and knockdown efficiency was quantified by RT-qPCR. (**B**-**C**) Immunostaining was conducted under different conditions as indicated and percentage of cells with mRFP-DCP1a foci was quantified. *P*-value in panel **A** was calculated by Student’s unpaired *t*-test. All the white scale bars correspond to 10μm. n.d. = not detected.

**Supplemental Figure 7. Antiviral activity of XRN1-DCP1/2 is independent of TBK1, MAVS, STING and NFκB.**

(**A**) Schematic diagram for indicated siRNA co-transfection experiments. Knockdown efficiency of each target was measured by qRT-PCR. The one with the best knockdown efficiency was used in subsequent study. (**B**) U138 cells treated as in (**A**) were infected with NDV (MOI=1). Levels of *NDV N* mRNA were measured using qPCR. (**C**) HeLa cells were transfected with indicated siRNAs for 48 hr, followed by NDV (MOI=1) infection. Cells were immunostained with indicated antibodies. White box for a specific region was enlarged. Cells with XRN1 foci were quantified and presented in the right panel. (**D**) HeLa cells were transfected with indicated siRNAs for 48 hr. Cells were then transfected with poly(I:C) (0.5µg) for 12 hr, protein lysates were collected and subjected to immunoblotting analysis with the indicated antibodies (*upper*). An independent set of control experiments with similar condition was prepared to evaluate siRNA knockdown efficiency (*lower*). (**E**) HEK293T cells were co-transfected with NFκB, pRL-TK reporter constructs (0.1µg), together with indicated siRNAs for 48 hr. Cells were then either mock-infected, or treated with TNFα (10ng/mL), LPS (100ng/mL) or RNAi-poly(I:C) (0.5µg). Cells were lysed to measure the luciferase activities. (**F**) MDA5-*Crispr/Cas9* deficient HeLa cells were transfected with indicated siRNAs for 48 hr, followed by treatment with either DMSO (vehicle), BAY 11-7082 (10µM), or TPCA1 (1.0µM), and subjected to CVB3 (MOI=1) infection for 4 hr. Levels of CVB3 *VP1* mRNA were measured by qPCR. (**G**) HeLa cells were subjected to similar condition as described in panel (**F**), and immunostained with antibody against endogenous XRN1 (green), and DAPI (blue) as nuclear marker. Orange box dotted line for a specific region was enlarged. Percentage of cells with XRN1 foci was quantified. White bars correspond to 10µm. (Statistical analyses in **A**, **B**, **C**, **D**, **F** and **G** are unpaired Student’s *t* test).
