# Supplementary figures and images for: Spatio-temporal characterization of the antiviral activity of the XRN1-DCP1/2 aggregation against cytoplasmic RNA viruses to prevent cell death
Source: Cell Death Differ. 2020 Feb 7;27(8):2363–82. doi: 10.1038/s41418-020-0509-0 (PMC7370233; doi:10.1038/s41418-020-0509-0)

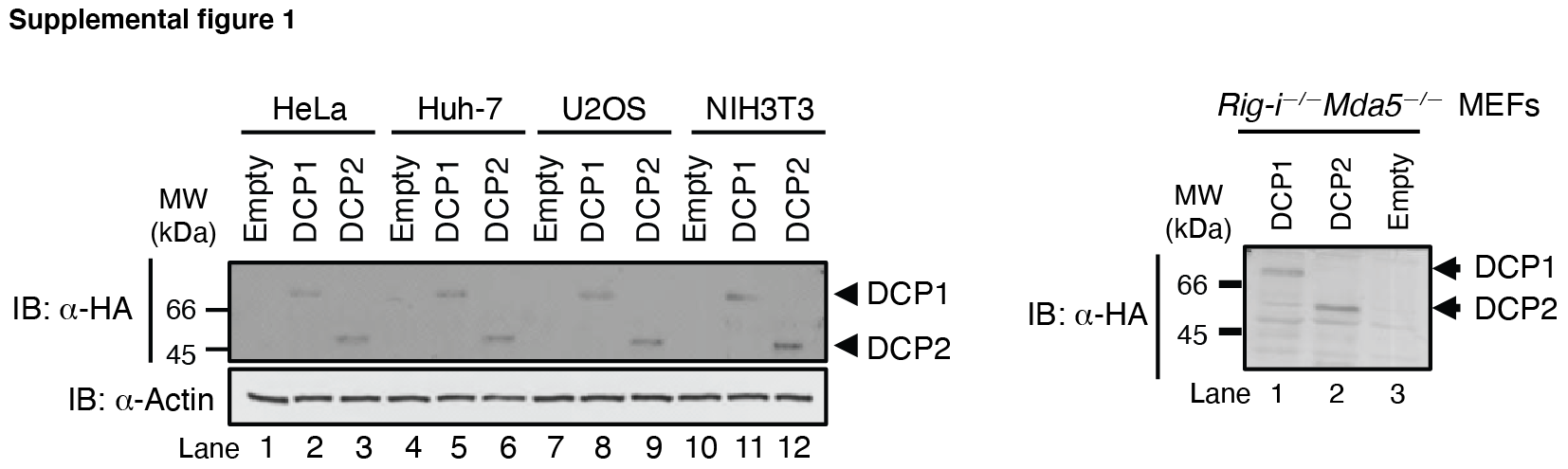

Supplement: Supplementary file 2 — Supplemental Figure 1 [file 41418_2020_509_MOESM2_ESM.png]

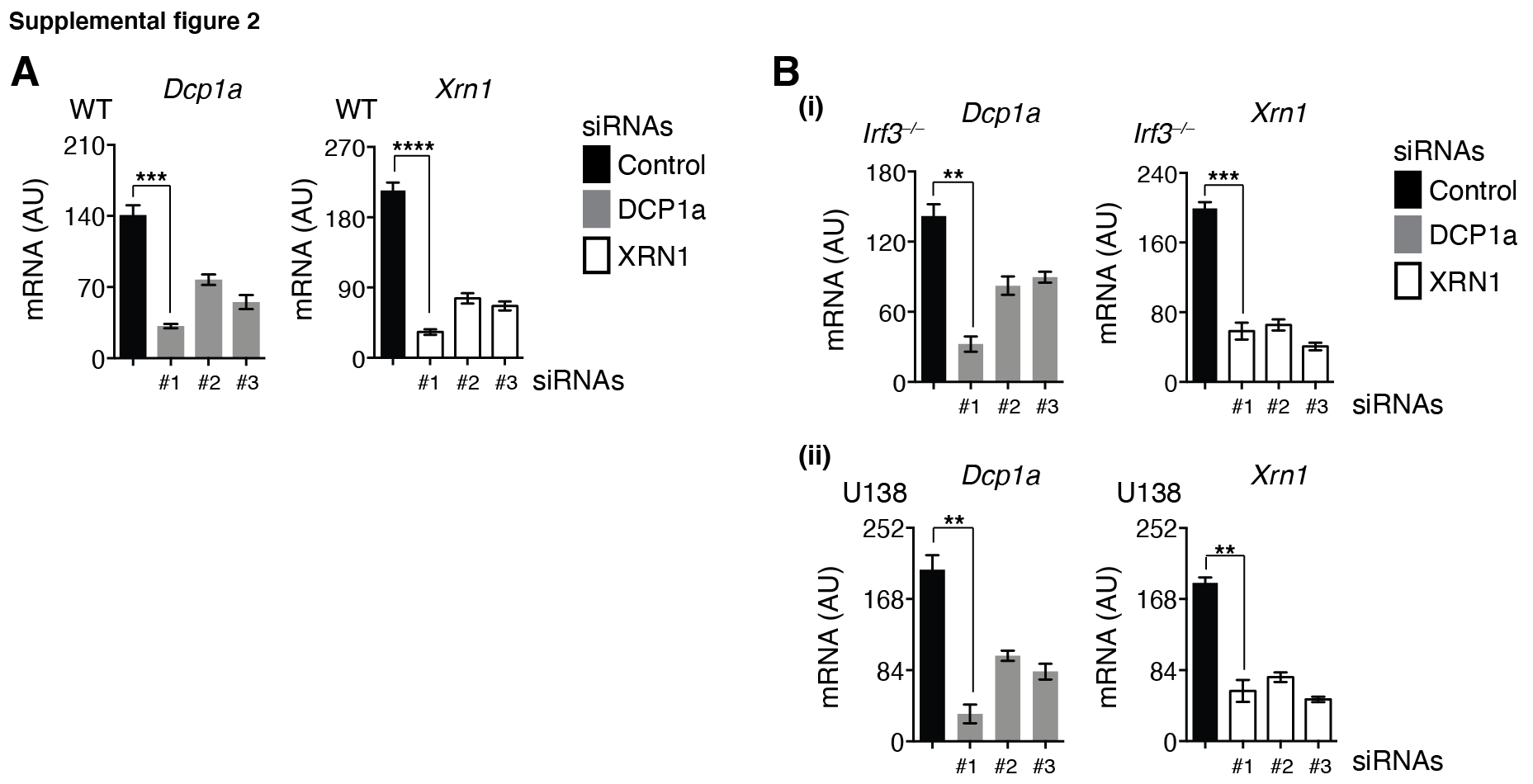

Supplement: Supplementary file 3 — Supplemental Figure 2 [file 41418_2020_509_MOESM3_ESM.png]

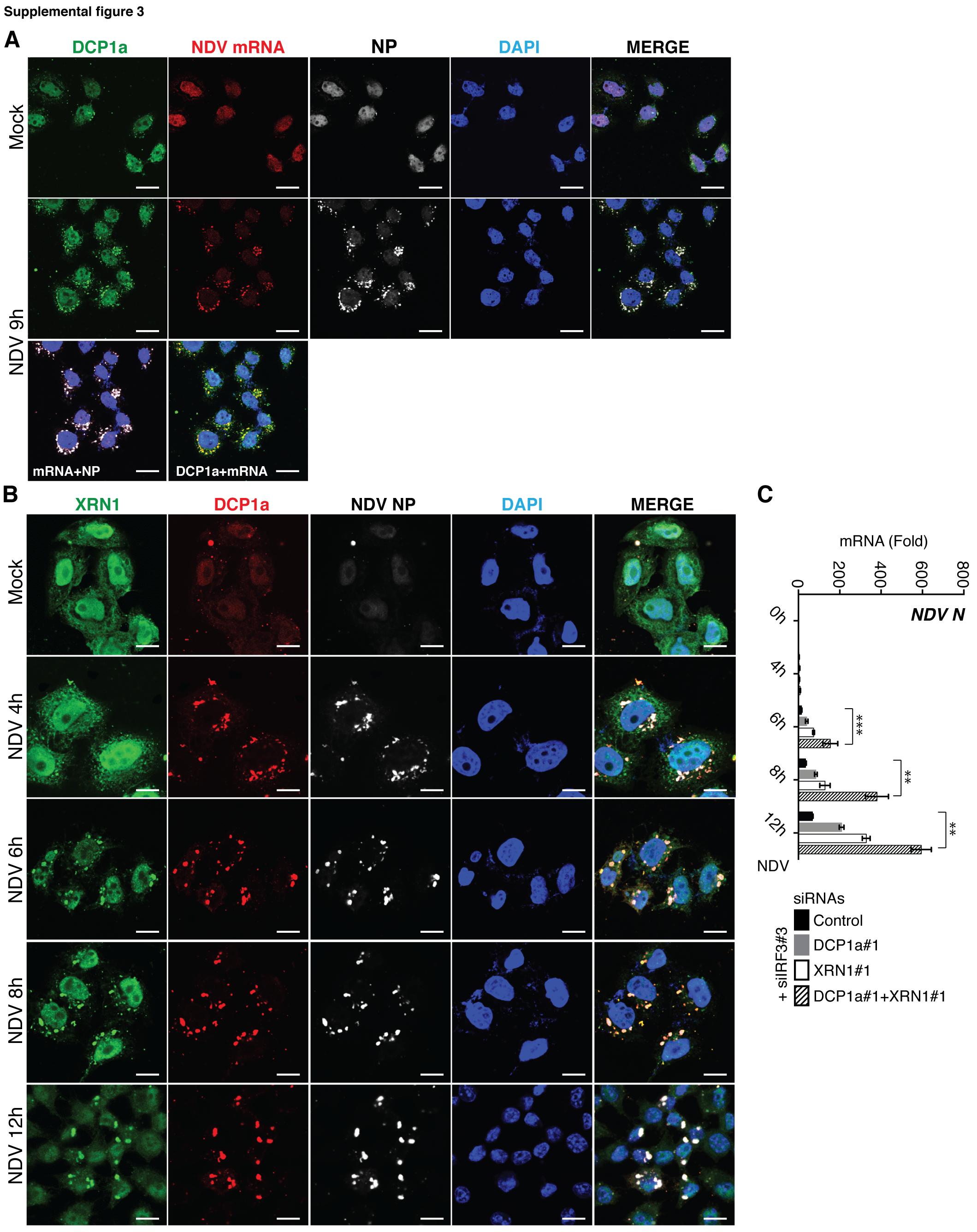

Supplement: Supplementary file 4 — Supplemental Figure 3 [file 41418_2020_509_MOESM4_ESM.png]

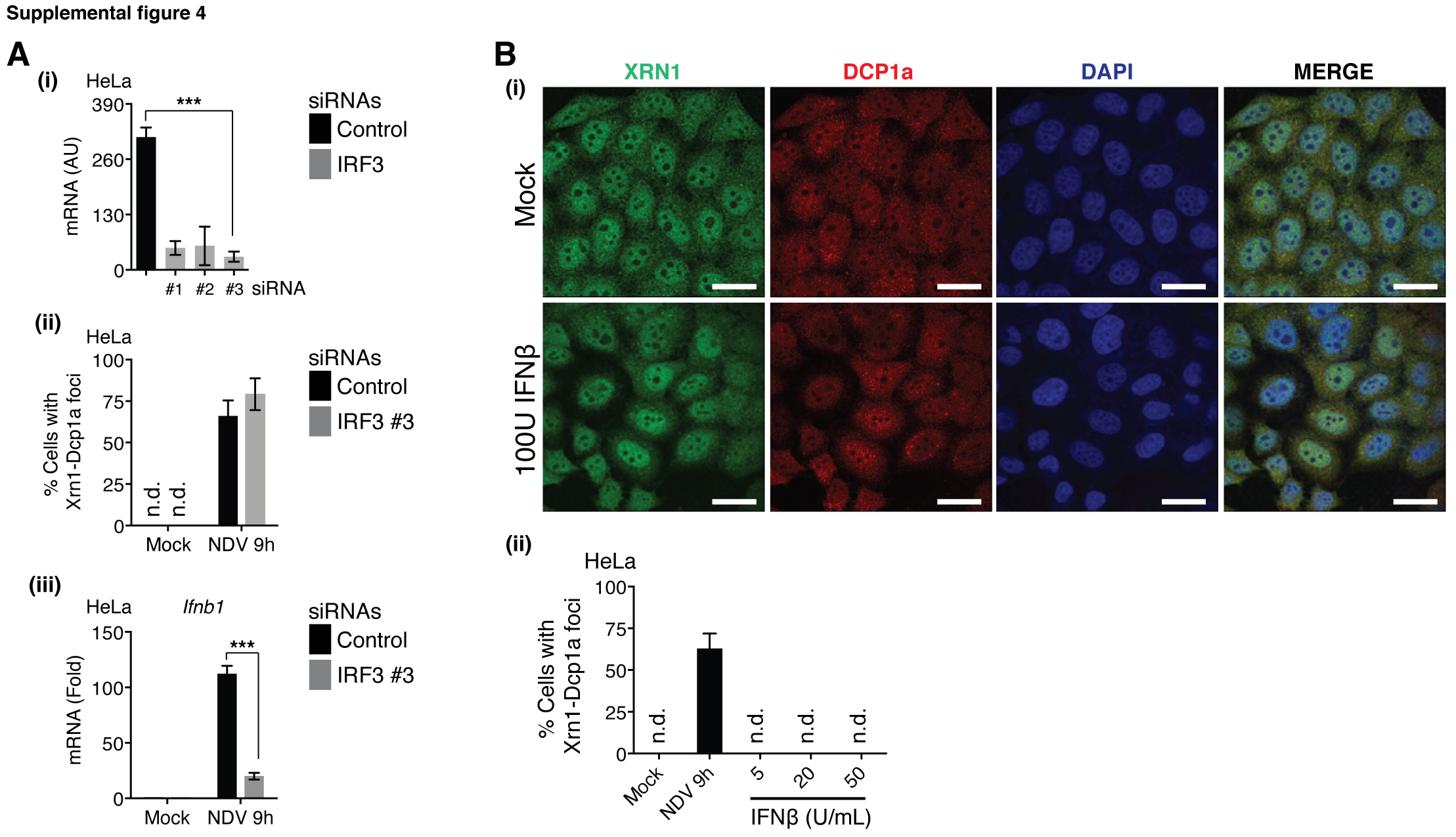

Supplement: Supplementary file 5 — Supplemental Figure 4 [file 41418_2020_509_MOESM5_ESM.png]

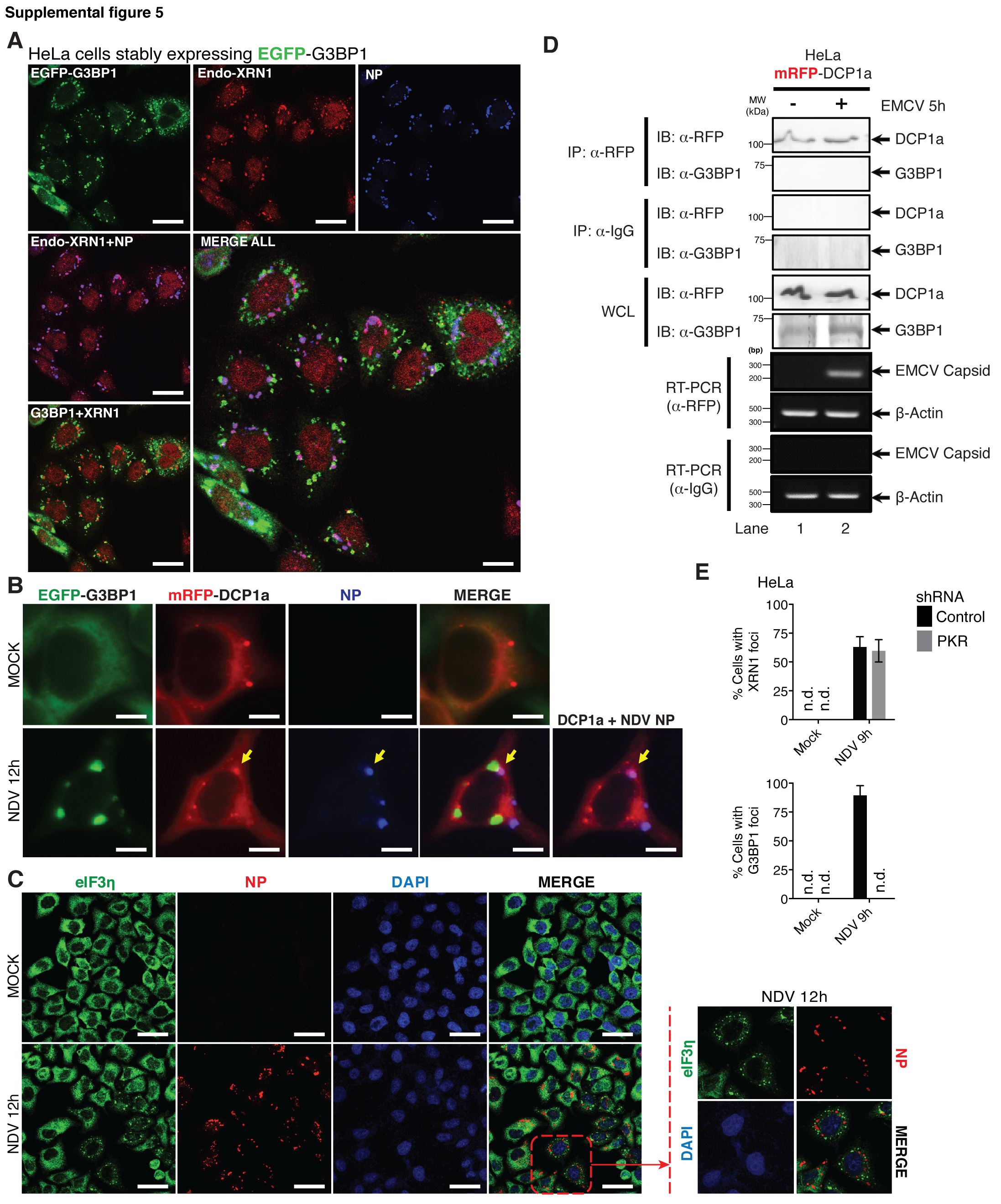

Supplement: Supplementary file 6 — Supplemental Figure 5 [file 41418_2020_509_MOESM6_ESM.png]

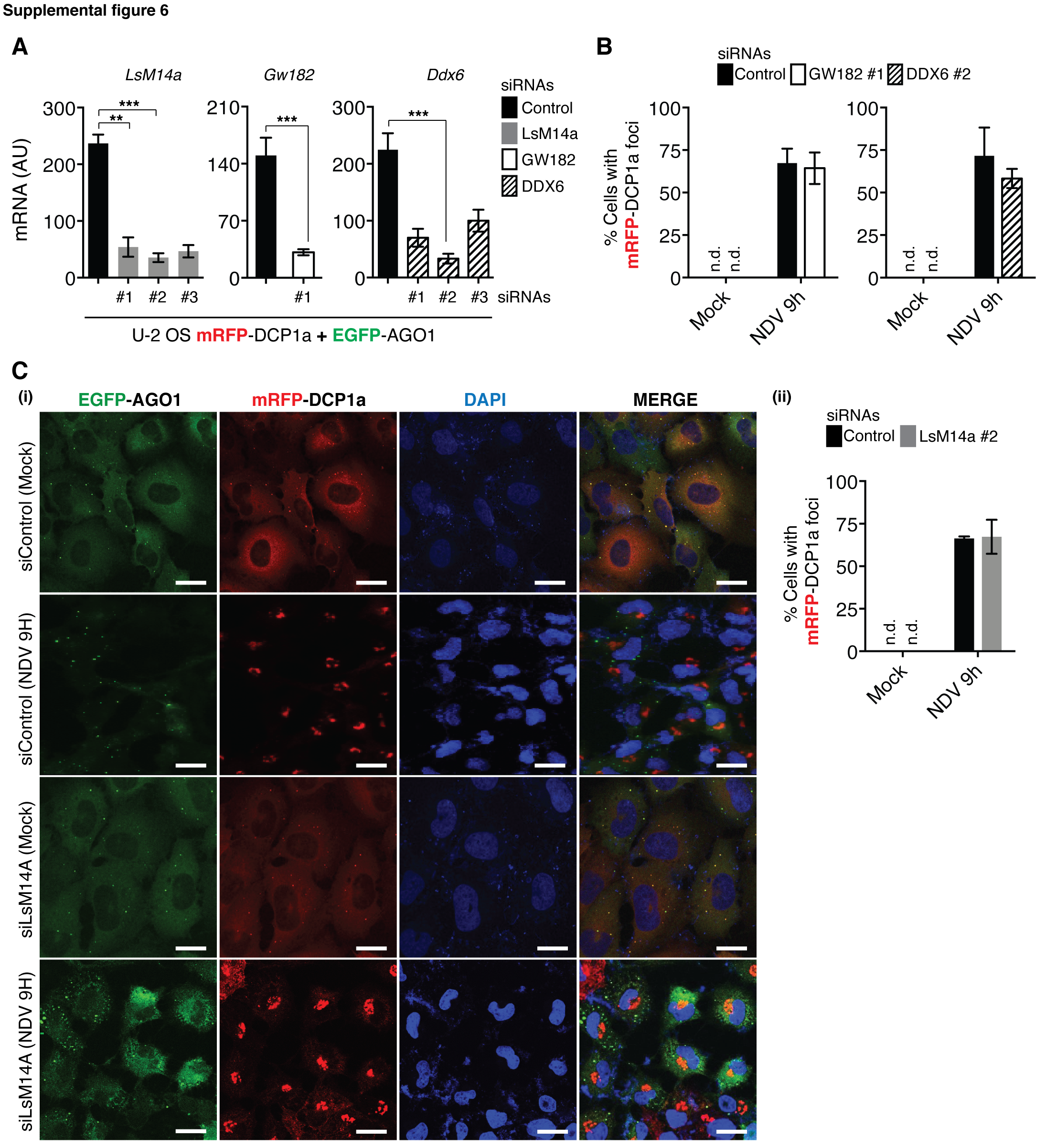

Supplement: Supplementary file 7 — Supplemental Figure 6 [file 41418_2020_509_MOESM7_ESM.png]

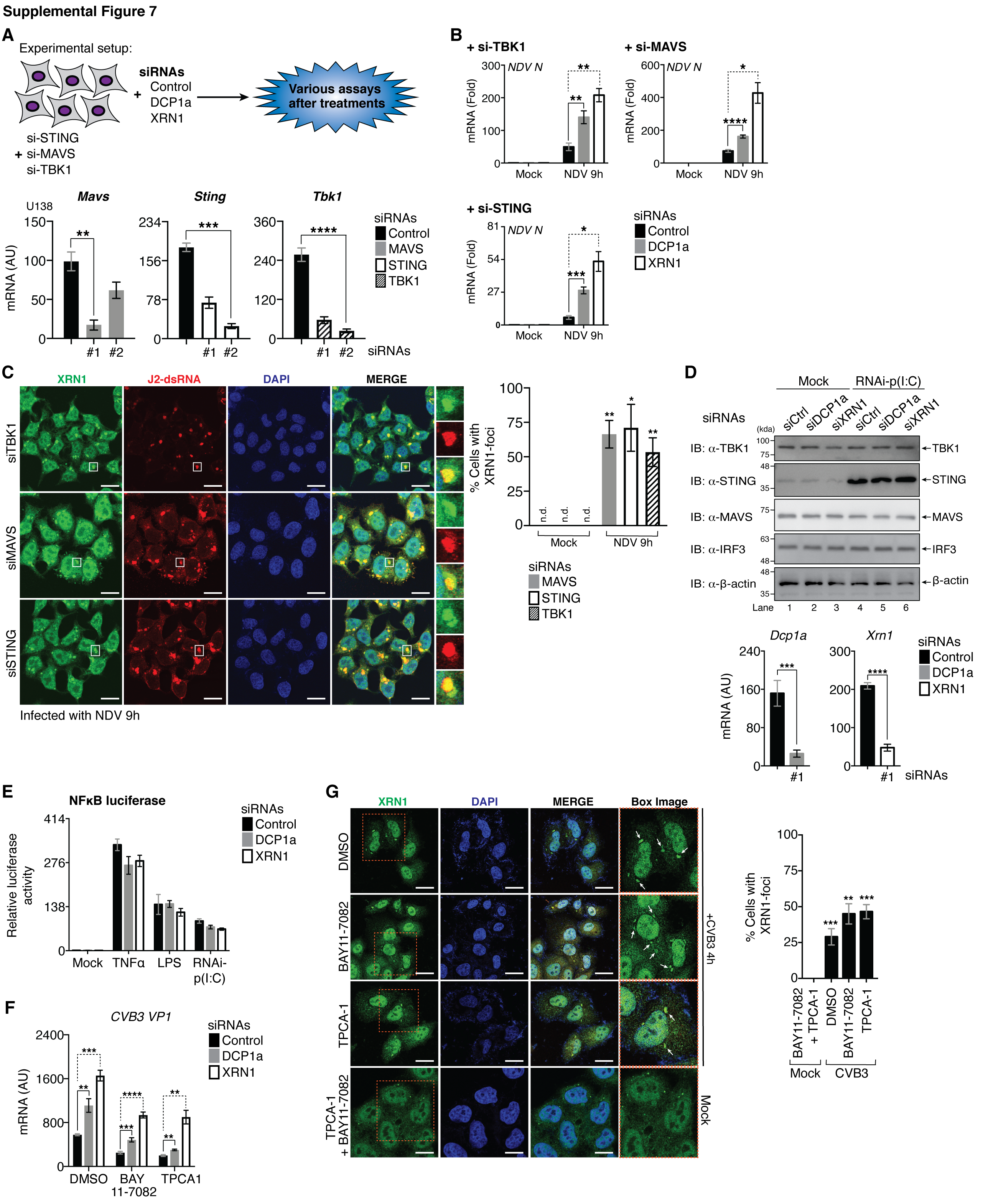

Supplement: Supplementary file 8 — Supplemental Figure 7 [file 41418_2020_509_MOESM8_ESM.png]
